# Supplementary figures and images for: Significant sparse polygenic risk scores across 813 traits in UK Biobank
Source: PLoS Genet. 2022 Mar 24;18(3):e1010105. doi: 10.1371/journal.pgen.1010105 (PMC8946745; doi:10.1371/journal.pgen.1010105)

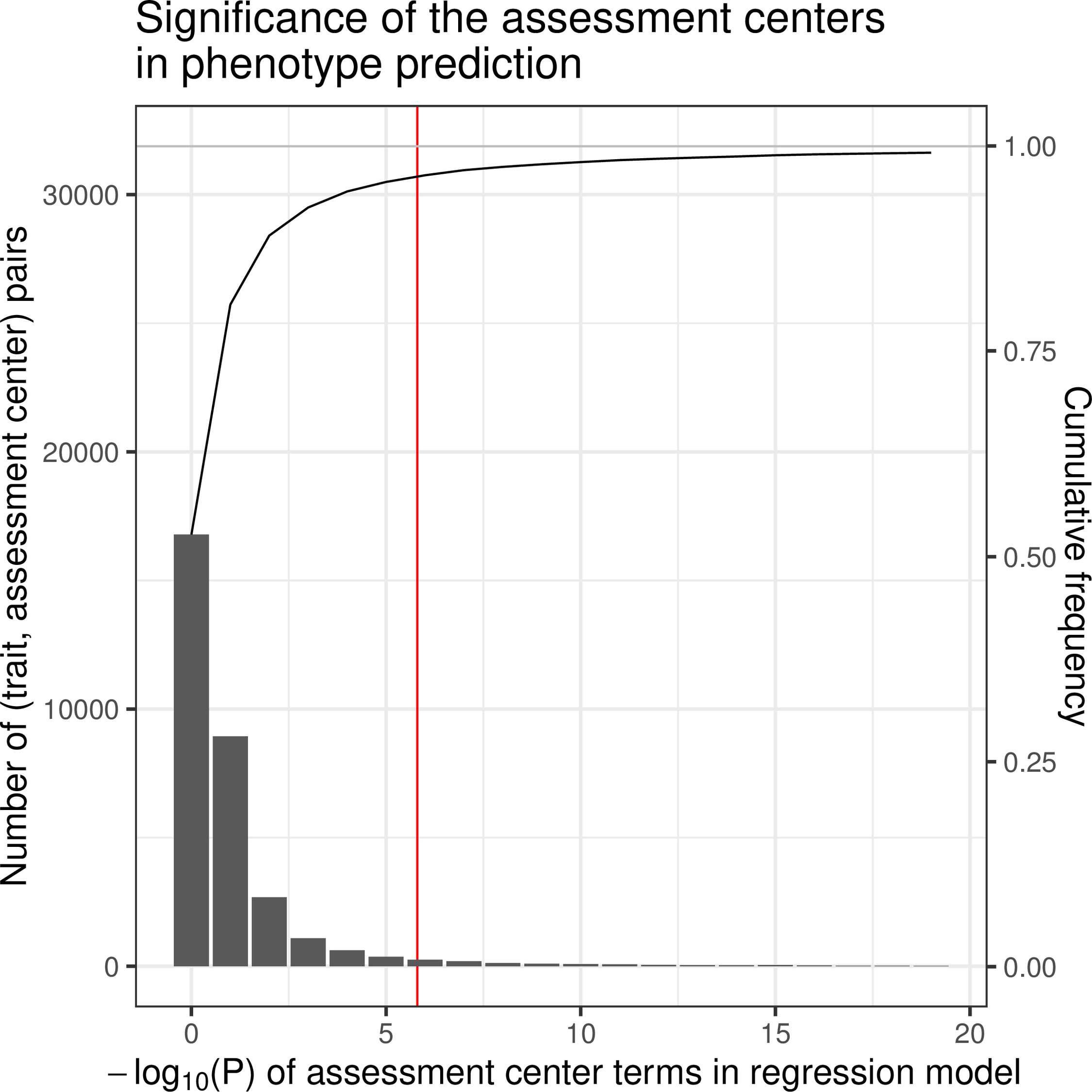

Supplement: S1 Fig — We fit a regression model on age, sex, the types of genotyping arrays, polygenic risk score, and assessment centers for each of the 1,565 traits analyzed in the study. The frequency of the statistical significance (-log10(P)) of assessment center variables was shown. The cumulative frequency was shown on the secondary axis on the right. The statistical significance after the Bonferroni correction was shown as a red vertical line. (TIF) [file pgen.1010105.s001.tif]

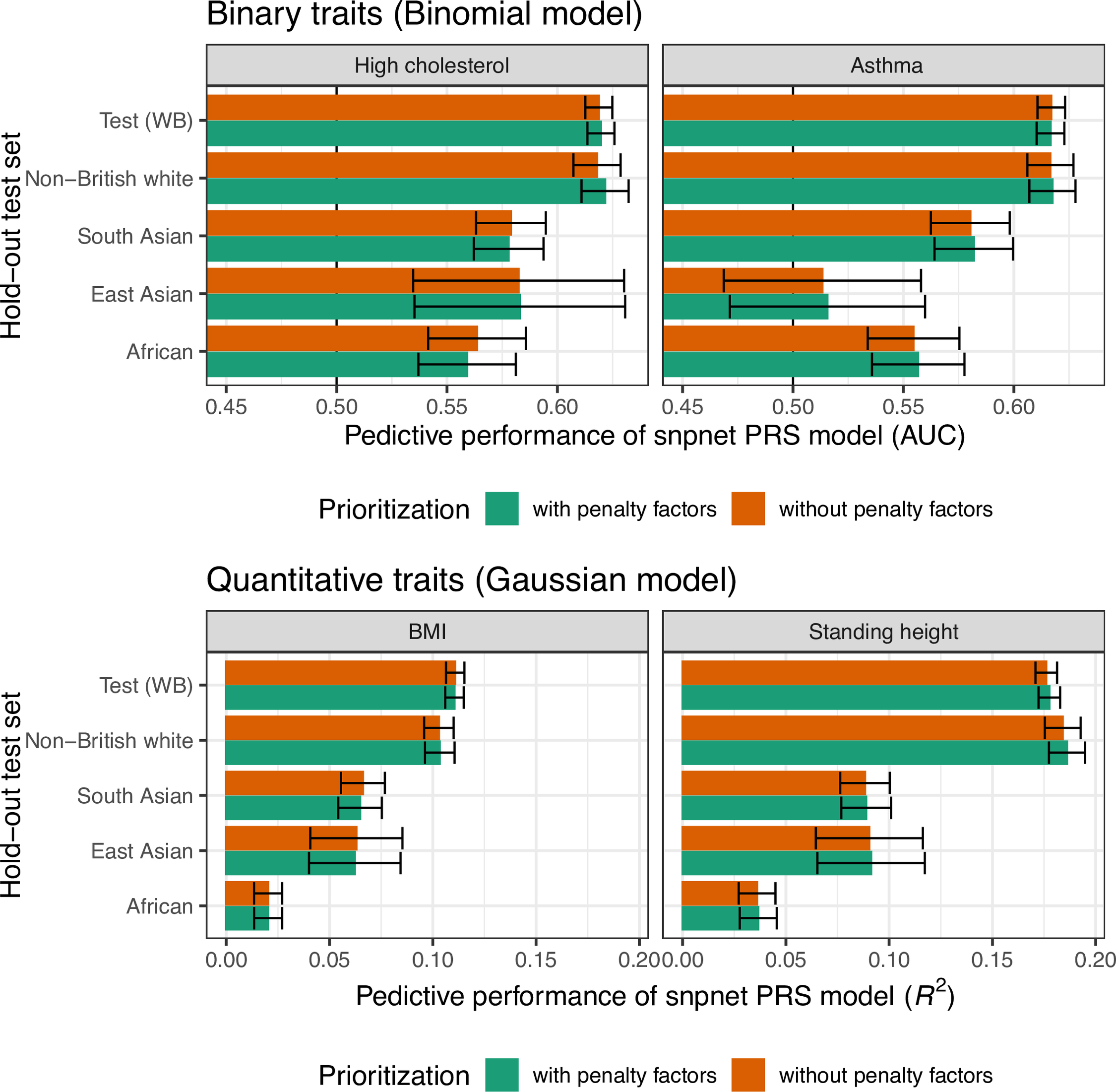

Supplement: S2 Fig — The predictive performance (AUC for binary traits and R2 for quantitative traits) evaluated across hold-out test set individuals of different ancestry groups in UK Biobank are shown for four traits. The error bars represent the 95% confidence interval. (TIF) [file pgen.1010105.s002.tif]

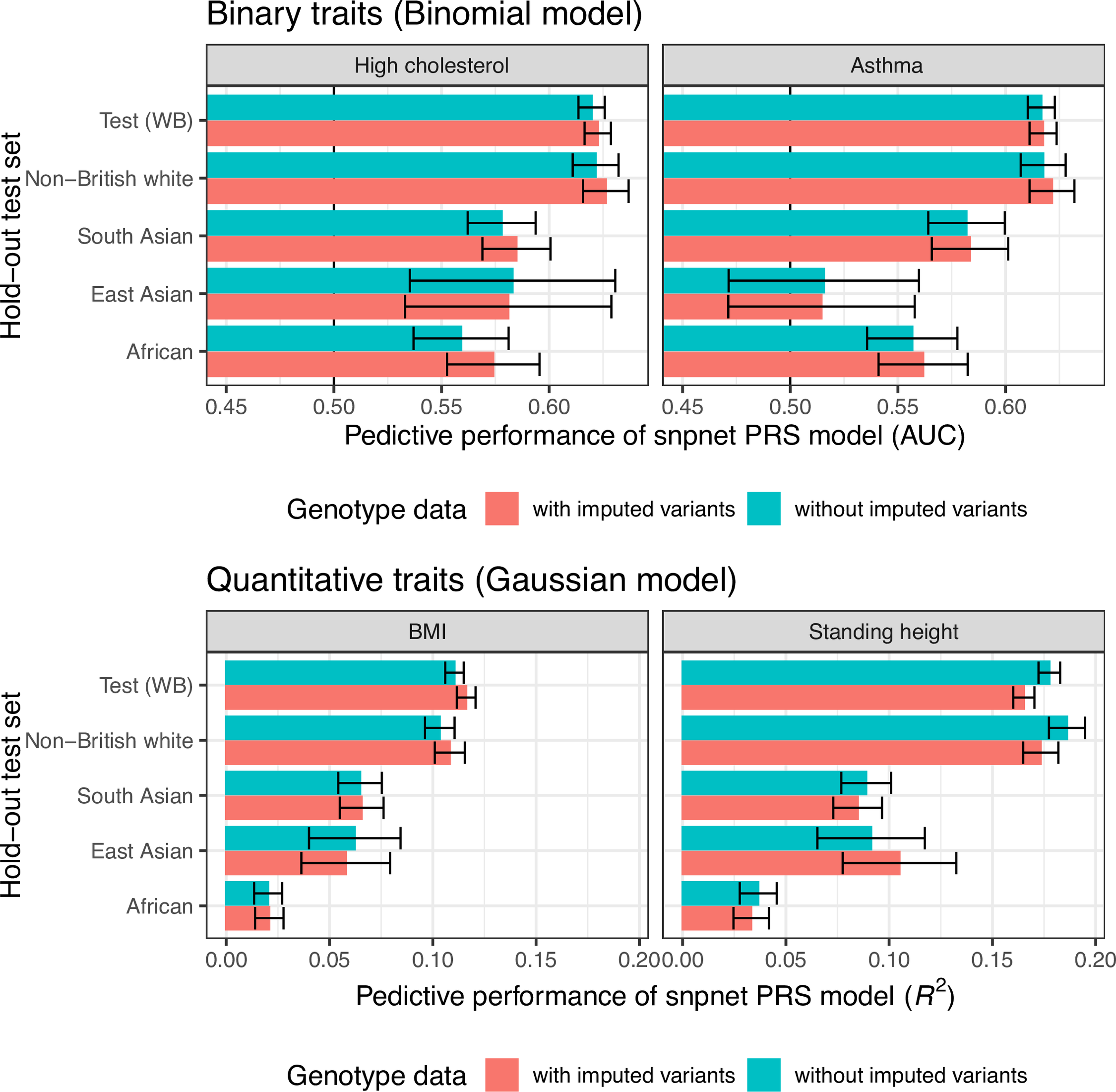

Supplement: S3 Fig — The predictive performance (AUC for binary traits and R2 for quantitative traits) evaluated across hold-out test set individuals of different ancestry groups in UK Biobank are shown for four traits. The error bars represent the 95% confidence interval. (TIF) [file pgen.1010105.s003.tif]
