# Supplementary material for: Significant sparse polygenic risk scores across 813 traits in UK Biobank
Source: PLoS Genet. 2022 Mar 24;18(3):e1010105. doi: 10.1371/journal.pgen.1010105 (PMC8946745; doi:10.1371/journal.pgen.1010105)
Supplement: Attachment — Submitted filename: PRSmap 20211109 1140 response to reviewers.pdf [file pgen.1010105.s011.pdf]

Please find the review response and revision regarding our manuscript “Significant Sparse Polygenic Risk Scores across 813 traits in UK Biobank” (PGENETICS-D-21-01210). Our responses to the reviewers’ individual comments below are in blue font, the comments from the reviewer are copied in black, and quoted texts from the updated manuscript are shown in gray with a vertical bar (examples are shown below):

This is an example of the reviewer’s comments
This is an example of our response.

> *This is an example of quoted texts from the updated manuscript*

Based on the feedback from the reviewers, we made significant updates to the manuscript. The major points of updates are as follows:

- The list of traits analyzed in the study. In the original manuscript, we have included 1,617 traits but in the latest version of the manuscript, we present the results for 1,565 traits. We realized that there were some issues in the trait definition and needed to revise the list.
- The number of significant PRS models. In the original manuscript, we reported the significant PRS models for 428 traits. While we were preparing the revision of the manuscript, we realized that there was a critical mistake when evaluating the significance of the PRS models (specifically, the coefficients of covariates were mistakenly estimated on the test set individuals, not on the score development set). We fixed this issue and now present 813 significant PRS models in the latest version of the manuscript. We sincerely apologize for our mistake and inconvenience in the review process.
- Following the suggestions by reviewer #2, we added a new figure (**Fig 2**) comparing the estimated SNP-based heritability vs. the predictive performance of PRS models. For this figure, we computed pseudo-R^2 for binary traits.
- We added a few additional analyses, including the effects of the UK Biobank assessment center in the predictive performance (**S1 Fig**), effects of prioritization of medically-relevant alleles (**S2 Fig**), and comparison of predictive performance when we include the imputed variants (**S3 Fig**).

Our specific point-to-point responses to the reviewer’s comments are listed below.

Referee #1:

In this paper, the authors have applied BASIL, a method previously developed by the authors, to 1600 traits in the UK Biobank. They have also provided a Global Biobank Engine which shows the predictive power of their PRS. However, given the current state of this paper, I cannot recommend this for publishing, and here are my reasons:

Thank you very much for taking the time to review the manuscript and for providing detailed feedback. We are confident that your comments have improved the clarity of the manuscript. The summary of the key changes in this revision is summarized on the first page of this document. Here are the responses to your suggestions.

1. Most of the methods were "described elsewhere", reading the current paper, it is not possible for readers to know how exactly the PRS were calculated. It is also unclear how the authors incorporate the HLA allelotype, CNV data and how the penalty factors were applied to the BASIL model.

Thank you for encouraging us to improve the method section so that the descriptions are self-contained. Following your advice, we added the following descriptions:

Lines 405-424, Pages 15-16, Population stratification and genetic data, Methods

> *Using a combination of genotype principal components (PCs), the self-reported ancestry (UK Biobank Field ID 21000, https://biobank.ndph.ox.ac.uk/ukb/field.cgi?id=21000), and "in_white_British_ancestry_subset" column in the sample QC file from UK Biobank, we subsequently focused on people of self-identified white British (n = 337,129), self-identified non-British white (n = 24,905), African (n = 6,497), South Asian (n = 7,831), and East Asian (n = 1,704) ancestry as described elsewhere[23]. Briefly, we used a two-step procedure to define the five groups. We first used the genotype principal component loadings of the individuals and set thresholds on component 1 and component 2 as follows: (1) self-identified White British: -20 ≤ PC1 ≤ 40 and -25 ≤ PC2 ≤ 10 and in_white_British_ancestry_subset == 1; (2) self-identified non-British White: -20 ≤ PC1 ≤ 40, -25 ≤ PC2 ≤ 10, has a self-reported ancestry of White, and does not identify themselves as White British; (3) African: 260 ≤ PC1, 50 ≤ PC2, and does not identify themselves as any of the following: Asian, White, Mixed, or Other population groups; (4) South Asian: 40 ≤ PC1 ≤ 120, -170 ≤ PC2 ≤ -80, and does not identify themselves as any of the following: Black, White, Mixed, or Other population groups; and (5) East Asian: 130 ≤ PC1 ≤ 170, PC2 ≤ -230, and does not identify themselves as any of the following: Black, White, Mixed, or Other population groups. To refine the population definition by removing the outliers, we computed population-specific genotype PCs using approximately LD independent (R^2 < 0.5) common (population-specific minor allele frequency > 5%) biallelic variants outside of the major histocompatibility complex region[23]. We applied following thresholds[23]: (1) South Asian: -0.02 ≤ population-specific PC1 ≤ 0.03, -0.05 ≤ population-specific PC2 ≤ 0.02; and (2) East Asian: -0.01 ≤ population-specific PC1 ≤ 0.02, -0.02 ≤ population-specific PC2 ≤ 0.*

Lines 440-448, Page 16, Variant quality control and variant annotation, Methods

We performed variant quality control as described elsewhere[23,47,56]. Briefly, we focused on the variants passing the following criteria: (1) outside of the major histocompatibility complex (MHC) region (hg19 chr6:25477797-36448354); (2) the missingness of the variant is less than 1%, considering that the two genotyping arrays (the UK BiLEVE Axiom array and the UK Biobank Axiom array) cover a slightly different set of variants[19]; (3) the minor-allele frequency is greater than 0.01%; (4) Hardy-Weinberg disequilibrium test p-value is less than 1.0×10^{-7}; (5) Passed the comparison of minor allele frequency with the gnomAD dataset (version 2.0.1) as described before[47,50]; (6) We manually investigated the cluster plots for a subset of variants and removed 11 variants that have unreliable genotype calls[47].

Following your advice, we expanded the description of how we constructed the sparse PRS models. Of note, we clarified that the BASIL algorithm implemented in the R *snpnet* package takes individual-level data, not the GWAS summary statistics, as the input and the hold-out test set was used in the evaluation of the predictive performance.

Lines 495-515, Pages 17-18, Methods

Construction of sparse PRS models

Using the batch screening iterative Lasso (BASIL) algorithm implemented in the R snpnet package[10], we constructed the sparse PRS models for the 1,565 traits. We used the Gaussian family and the R2 metric for quantitative traits, whereas we used the binomial family and the AUC-ROC metric for the binary traits[10]. For each trait, we fit a series of regression models with a varying degree of sparsity on the training set, consisting of 70% (n = 235,991) of unrelated individuals of white British ancestry. The predictive performance of each of the models is evaluated on the validation set, which consists of 10% (n = 33,713) of unrelated individuals of white British ancestry to guide the selection of the optional level of sparsity. We selected the sparsity that maximizes the predictive performance in the validation set. We subsequently refit the penalized regression model using the individuals in the combined training and validation set individuals (n = 269,704), which we denote as score development set, to maximize the power in the regression model[10]. We used the same training, validation, and test set split across all the PRS models analyzed in this study.

As opposed to many PRS methods that operate on the GWAS summary statistics[3–9,13–15], our method takes individual-level genotype and phenotype data. Using L1 penalized regression (also known as Lasso), BASIL simultaneously performs variable selection and effect size estimation of the selected variants. We included age, sex, and top ten population-specific genotype PC loadings computed for the white British individuals[23] as unpenalized covariates. Thanks to the L1 penalty term in the objective function that penalizes the number of features of non-zero regression coefficients, the resulting models will be sparse, meaning that they will have fewer genetic variants than unpenalized models[10].

Lines 526-531, Page 18, Methods

> ***Predictive performance and transferability of PRS models***
>
> *We evaluated the predictive performance (R^2 for quantitative traits and receiver operating characteristic area under the curve [ROC-AUC] for binary traits) of PRS models. We used the individuals in the hold-out test set (n = 67,425) of white British ancestry as well as additional sets of individuals in non-British white (n = 24,905), African (n = 6,497), South Asian (n = 7,831), and East Asian (n = 1,704) ancestry groups.*

Following your advice, we expanded our description on the copy number variants (CNVs) and HLA allelotypes used in the analysis.

Lines 455-463, Page 16, Variant quality control and variant annotation, Methods

> *We included the imputed copy number variants (CNVs)[22] and imputed HLA allelotypes[21]. The CNVs were called using PennCNV (v.1.0.4)[60] on raw signal intensity data from each genotyping array as described elsewhere[22]. Because the precise location of the CNVs is not identified, we did not infer the functional consequences of CNVs with variant annotation. The HLA allelotypes at HLA-A, -B, -C, -DPA1, -DPB1, -DQA1, -DQB1, -DRB1, -DRB3, -DRB4, and -DRB5 loci were imputed using the HLA*IMP:02 and imputed dosage file is provided by the UK Biobank. We included 156 alleles across all 11 loci that had a frequency of 0.1% or greater in the white British. We rounded allele dosage when they were within plus or minus 0.1 of 0, 1, or 2. We excluded the remaining nonzero entries. We also excluded erroneous total allele counts post-rounding[21].*

We have also expanded our description on the derivation of the penalty factors. Of note, we added two supplementary tables (**S4 Table and S5 Table**) to improve the clarity of the description.

Lines 430-438, Page 16, Methods

> ***Variant quality control and variant annotation***
>
> *We used genotype datasets (release version 2 for the directly genotyped variants and the imputed HLA allelotype datasets)[19], the CNV dataset[22], and the hg19 human genome reference for the main PRS analyses in the study. Additionally, we considered imputed variants (release version 3) to investigate whether the imputed variants would improve the predictive performance. We annotated the directly-genotyped variants using Ensembl's Variant Effect Predictor (VEP) (version 101)[58,59] with the LOFTEE plugin (https://github.com/konradjk/loftee)[50], for which we created a Docker container image (https://github.com/yk-tanigawa/docker-ensembl-vep-loftee). Using ClinVar (version 20200914)[28], we annotated "pathogenic" and "likely pathogenic" variants.*

Lines 450-453, Page 16, Variant quality control and variant annotation, Methods

> *We grouped the VEP-predicted consequence of the variants into six groups: protein-truncating variants (PTVs), protein-altering variants (PAVs), proximal coding variants (PCVs), Intronic variants*

(Intronic), variants in the untranslated region (Intronic), and other variants (Others). Our grouping rule of the VEP-predicted consequence is summarized in (S4 Table).

Lines 517-525, Page 18, Construction of sparse PRS models, Methods

To prioritize coding variants over non-coding variants in linkage, we assigned three levels of penalty factors (also known as penalty scaling parameter)[62]: 0.5 for pathogenic variants in ClinVar[53] or protein-truncating variants according to VEP-based variant annotation[59]; 0.75 for likely pathogenic variants in ClinVar, VEP-predicted protein-altering variants, or imputed allelotypes; and 1.0 for all other variants. The assignment rules of the penalty factors are summarized in (S5 Table). The variants with lower values of penalty factors are prioritized in the L1 penalized regression. To assess the degree of prioritization of the medically relevant alleles and their impacts on the predictive performance, we focused on four traits (standing height, BMI, high cholesterol, and asthma) and fit a separate model without penalty factors. We compared the number of selected variants and the predictive performance.

Thank you very much for your suggestion.

2. In addition, the authors did not provide any details of how they obtained the GWAS summary statistics required for PRS calculation (presumably using the 70% UK biobank data using PLINK?)

Thank you very much for clarifying this. As described in the previous item, the BASIL algorithm implemented in the R *snpnet* package takes individual-level data, not the summary statistics. We agree that this could be a potential source of confusion to the readers who are familiar with the PRS methods that take GWAS summary statistics. We updated our description.

Lines 495-515, Pages 17-18, Methods

Construction of sparse PRS models

Using the batch screening iterative Lasso (BASIL) algorithm implemented in the R snpnet package[10], we constructed the sparse PRS models for the 1,565 traits. We used the Gaussian family and the R2 metric for quantitative traits, whereas we used the binomial family and the AUC-ROC metric for the binary traits[10]. For each trait, we fit a series of regression models with a varying degree of sparsity on the training set, consisting of 70% (n = 235,991) of unrelated individuals of white British ancestry. The predictive performance of each of the models is evaluated on the validation set, which consists of 10% (n = 33,713) of unrelated individuals of white British ancestry to guide the selection of the optional level of sparsity. We selected the sparsity that maximizes the predictive performance in the validation set. We subsequently refit the penalized regression model using the individuals in the combined training and validation set individuals (n = 269,704), which we denote as score development set, to maximize the power in the regression model[10]. We used the same training, validation, and test set split across all the PRS models analyzed in this study.

As opposed to many PRS methods that operate on the GWAS summary statistics[3–9,13–15], our method takes individual-level genotype and phenotype data. Using L1 penalized regression (also

known as Lasso), BASIL simultaneously performs variable selection and effect size estimation of the selected variants. We included age, sex, and top ten population-specific genotype PC loadings computed for the white British individuals[23] as unpenalized covariates. Thanks to the L1 penalty term in the objective function that penalizes the number of features of non-zero regression coefficients, the resulting models will be sparse, meaning that they will have fewer genetic variants than unpenalized models[10].

3. Usually, we would include the UK Biobank assessment centre as a covariate to UK Biobank related analysis to avoid systematic collection error. In addition, for blood biomarkers, we usually want to include Fasting time, dilution factor and statin use, as those usually have significant impact to the model fit.

Thank you very much for raising the points on covariate adjustment.

Regarding the assessment centers, we performed additional analysis to investigate whether the assessment center is significantly correlated with the phenotypes of our interest. As you may see below, we found the assessment center terms do not pass the significance threshold (after correcting it with Bonferroni). We, therefore, did not include the assessment center in the covariates. We updated the manuscript. Thank you for your suggestion.

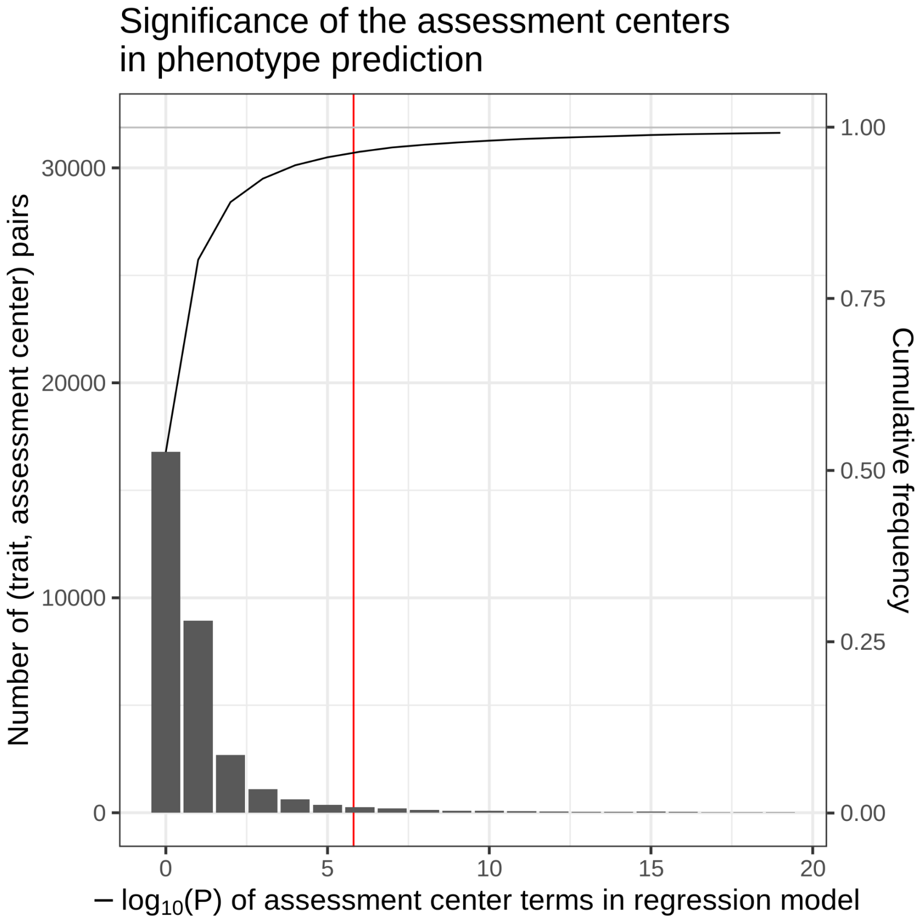

Fig S1. Statistical significance of the assessment center terms in phenotype prediction. We fit a regression

model on age, sex, the types of genotyping arrays, polygenic risk score, and assessment centers for each of the 1,565 traits analyzed in the study. The frequency of the statistical significance ($-\log_{10}(P)$) of assessment center variables was shown. The cumulative frequency was shown on the secondary axis on the right. The statistical significance after the Bonferroni correction was shown as a red vertical line.

Line 150-151, Page 5, Characterizing sparse PRS models with BASIL algorithm, Results

> *We found the identity of the UK Biobank assessment centers mostly has a non-significant impact on the predictive performance (S1 Fig, Methods).*

For the blood biomarker traits, we have previously investigated the effects of the covariates and derived covariate-adjusted biomarker values (Sinnott-Armstrong, *et al*. 2021, PMID: 33462484). There, we found that most of the 35 blood and urine biomarker traits are quite correlated between the raw biomarker levels and the fully adjusted values (Pearson's correlation ~0.95). Of note, after adjustments for age and sex, most results will remain interpretable without adjusting for additional variables.

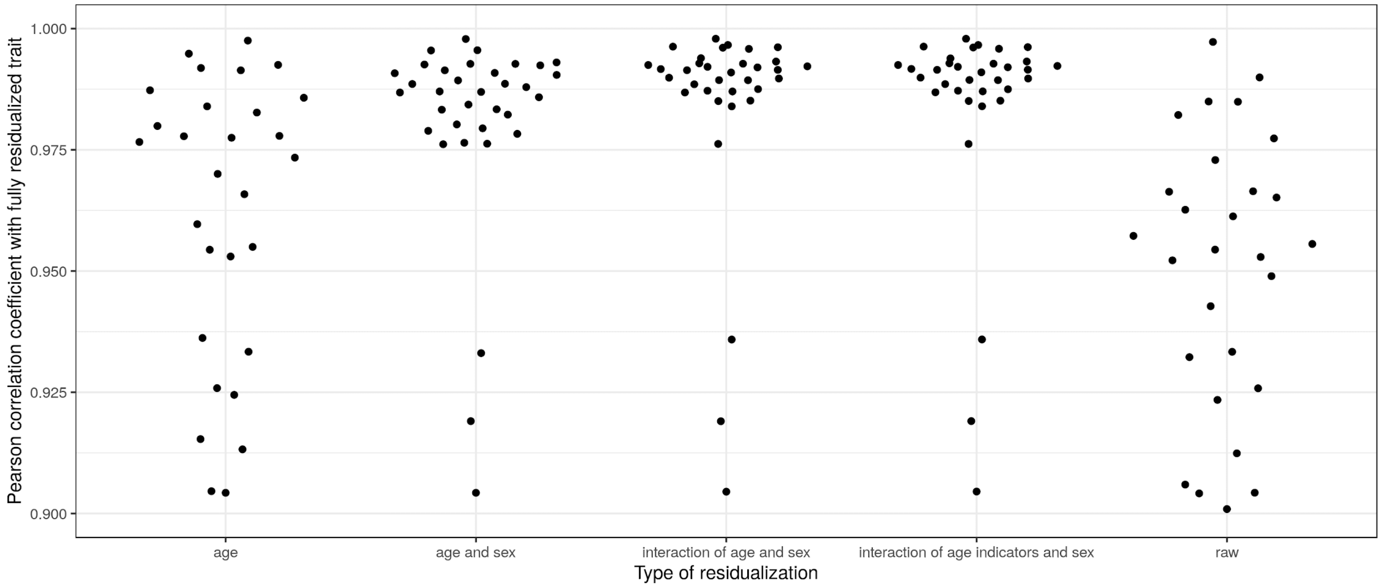

Figure. The effects of covariates on blood and urine biomarker levels. We adjusted technical and biological covariates for blood and urine biomarkers analyzed in our previous study (Sinnott-Armstrong, *et al*. 2021, PMID: 33462484). The Pearson's correlation between the fully-adjusted models and partially-adjusted (or the raw measurement values) are shown for 35 biomarker traits. The three outlier traits with lower correlations in the more adjustments are Vitamin D (for which month of assessment is quite important), Phosphate (for which assessment center is quite important), and Glucose (for which fasting time is quite important).

In the original version of the manuscript, we have included both the raw measured biomarker values and the covariate-adjusted biomarker values as separate traits and characterized the PRS model for

each of them. We apologize that this point was not clear in the original manuscript, which caused the confusion of the predictive performance of the covariate-only model for some traits (point #7).

We now removed those duplicated biomarker traits. Specifically, we used the PRS models trained on covariate-adjusted biomarker traits and evaluated their predictive performance using the raw phenotype values, with the exception of the following three traits: eGFR, AST/ALT ratio, and non-albumin protein. Those three phenotypes are derived based on a combination of covariate-adjusted other biomarker traits and do not have the raw measurements.

Line 480-491, Page 17, Phenotype definitions in the UK Biobank, Methods

> *Previously, we analyzed blood and urine biomarker traits, investigating the effects of covariates on the biomarker levels and derived covariate-adjusted biomarker values[23]. Briefly, we used a linear regression model to account for the covariate effects on the log-transformed measurement values from UK Biobank and adjusted for principal component loadings of genotype, age, sex, age by sex interactions, self-identified ancestry group, self-identified ancestry group by sex interactions, fasting time, estimated sample dilution factor, assessment center indicators, genotyping batch indicators, time of sampling during the day, the month of assessment, and day of the assay. We used the PRS models trained for the covariate-adjusted traits[23]. To quantify the incremental predictive performance against the covariate-only models, we quantified predictive performance against the original measurement values, except eGFR, AST/ALT ratio, and non-albumin protein, where we used the covariate-adjusted trait values. Those three traits are derived from covariate-adjusted biomarkers[23] and do not have raw measurement values.*

Thank you very much for giving us an opportunity to clarify the covariate handling.

4. Was the metric reported based on the test sets?

Yes, we used the hold-out test set when evaluating the performance metric. We improved the clarity on this throughout the manuscript.

Lines 81-83, Page 3, Introduction

> *Using individuals in a hold-out test set, we evaluated their predictive performance and their statistical significance, resulting in 813 significant ($p < 2.5 \times 10^{-5}$) PRS models*

Lines 135-142, Page 5, Characterizing sparse PRS models with BASIL algorithm, Results

> *To evaluate the predictive performance (R^2 for quantitative traits and receiver operating characteristic area under the curve [ROC-AUC] for binary traits) and its significance of PRS models, we focused on the remaining 20 % of unrelated individuals in the hold-out test set (n = 67,425) as well as additional sets of unrelated individuals in the following ancestry groups in UK Biobank: non-British European (non-British white, n = 24,905), African (n = 6,497), South Asian (n = 7,831), and East Asian (n = 1,704) (S2 Table, Methods). We found 813 PRS models with significant ($p < 2.5 \times 10^{-5} = 0.05/2,000$,*

adjusted for multiple hypothesis testing with Bonferroni method) predictive performance in the hold-out test set of white British individuals (Methods).

Lines 526-531, Page 18, Methods

Predictive performance and transferability of PRS models

We evaluated the predictive performance (R^2 for quantitative traits and receiver operating characteristic area under the curve [ROC-AUC] for binary traits) of PRS models. We used the individuals in the hold-out test set (n = 67,425) of white British ancestry as well as additional sets of individuals in non-British white (n = 24,905), African (n = 6,497), South Asian (n = 7,831), and East Asian (n = 1,704) ancestry groups.

5. How did the authors use the PCS and self-reported ancestry to identify the sample population? K mean clustering on PC1 and PC2? Or did they performed calculate the Euclidian distance between each sample and the PC centroid of each self-reported cluster?

Thank you very much for encouraging us to further clarify the population stratification procedure. Following your advice, we expanded the description in the methods section.

Lines 405-424, Pages 15-16, Population stratification, Methods

Using a combination of genotype principal components (PCs), the self-reported ancestry (UK Biobank Field ID 21000, https://biobank.ndph.ox.ac.uk/ukb/field.cgi?id=21000), and "in_white_British_ancestry_subset" column in the sample QC file from UK Biobank, we subsequently focused on people of self-identified white British (n = 337,129), self-identified non-British white (n = 24,905), African (n = 6,497), South Asian (n = 7,831), and East Asian (n = 1,704) ancestry as described elsewhere[23]. Briefly, we used a two-step procedure to define the five groups. We first used the genotype principal component loadings of the individuals and set thresholds on component 1 and component 2 as follows: (1) self-identified White British: -20 ≤ PC1 ≤ 40 and -25 ≤ PC2 ≤ 10 and in_white_British_ancestry_subset == 1; (2) self-identified non-British White: -20 ≤ PC1 ≤ 40, -25 ≤ PC2 ≤ 10, has a self-reported ancestry of White, and does not identify themselves as White British; (3) African: 260 ≤ PC1, 50 ≤ PC2, and does not identify themselves as any of the following: Asian, White, Mixed, or Other population groups; (4) South Asian: 40 ≤ PC1 ≤ 120, -170 ≤ PC2 ≤ -80, and does not identify themselves as any of the following: Black, White, Mixed, or Other population groups; and (5) East Asian: 130 ≤ PC1 ≤ 170, PC2 ≤ -230, and does not identify themselves as any of the following: Black, White, Mixed, or Other population groups. To refine the population definition by removing the outliers, we computed population-specific genotype PCs using approximately LD independent (R^2 < 0.5) common (population-specific minor allele frequency > 5%) biallelic variants outside of the major histocompatibility complex region[23]. We applied following thresholds[23]: (1) South Asian: -0.02 ≤ population-specific PC1 ≤ 0.03, -0.05 ≤ population-specific PC2 ≤ 0.02; and (2) East Asian: -0.01 ≤ population-specific PC1 ≤ 0.02, -0.02 ≤ population-specific PC2 ≤ 0.

6. Given the small sample size of non-European samples, does the author also split them into validation and test sets, or were the training all done on the validation sets? Based on page 10 line 230-242, I am guessing

that the GWAS is performed on the British white, and parameter optimization / variable selections were done on the British white and then the predictive performance is performed on each of the populations? Or were the parameter optimization / variable selections also done in each of the populations?

We used individual-level data of the unrelated individuals in the white British ancestry group to fit the PRS model. We evaluated the model using a hold-out test set of white British ancestry as well as additional test sets of different ancestry groups.

Lines 135-140, Page 5, Characterizing sparse PRS models with BASIL algorithm, Results

> *To evaluate the predictive performance (R^2 for quantitative traits and receiver operating characteristic area under the curve [ROC-AUC] for binary traits) and its significance of PRS models, we focused on the remaining 20 % of unrelated individuals in the hold-out test set (n = 67,425) as well as additional sets of unrelated individuals in the following ancestry groups in UK Biobank: non-British European (non-British white, n = 24,905), African (n = 6,497), South Asian (n = 7,831), and East Asian (n = 1,704) (S2 Table, Methods).*

Lines 526-531, Page 18, Methods

> ***Predictive performance and transferability of PRS models***
>
> *We evaluated the predictive performance (R^2 for quantitative traits and receiver operating characteristic area under the curve [ROC-AUC] for binary traits) of PRS models. We used the individuals in the hold-out test set (n = 67,425) of white British ancestry as well as additional sets of individuals in non-British white (n = 24,905), African (n = 6,497), South Asian (n = 7,831), and East Asian (n = 1,704) ancestry groups.*

We also included the study cohort information as a panel (A) in **Fig 1** and provided the basic characteristics of each set in **S1 Table**.

(A) Study cohort and phenotypes

378,066 unrelated individuals in UK Biobank (**S2 Table**)

337,129 individuals of white British ancestry
- score development (n = 269,704)
- hold-out test set (n = 67,425)

Additional hold-out sets for transferability assessment
- non-British white (n = 24,905)
- African (n = 6,497)
- South Asian (n = 7,831)
- East Asian (n = 1,704)

1,565 phenotypes in UK Biobank (**S1 Table**)

871 quantitative traits

Anthropometry, biomarkers, blood assays, bone densitometry ...

694 binary traits

Disease outcomes, cancer, lifestyle factors, family history ...

Figure 1. Significant sparse polygenic risk scores (PRSs) across 813 traits in the UK Biobank. (A) We analyzed a total of more than 378,000 unrelated individuals and 1,565 traits in UK Biobank. We used 80 % of individuals of white British ancestry for score development. For evaluation, we used the remaining 20 % of individuals and additional individuals in other ancestry groups.

7. Looking at the results shown in the Global Biobank Engine, there are many traits where the covariate has a predictive performance of 0 (assuming this is measured in R2). Specifically, for Lipoprotein A, its PRS performance is as high as 0.57 but the covariate has performance of 0, which is hard to believe.

Thank you very much for looking into the results on Global Biobank Engine.

As we explained in point #3 above, our original manuscript included covariate-adjusted biomarker traits from our previous study (Sinnott-Armstrong, *et al*. 2021, PMID: 33462484) and the predictive performance of the covariate-only model for those traits were zero. We agree that such treatment was inappropriate and have revised the analysis of biomarker traits. Specifically, we now have a non-redundant set of 35 biomarker traits. For eGFR, AST/ALT ratio, and non-albumin protein, which were all derived from other covariate-adjusted biomarker traits, we do not have the raw measurement and the predictive performances of the covariate-only models were zero. For the remaining 32 biomarker traits, we used the raw phenotypes for the performance evaluation.

On the Global Biobank Engine, the predictive performance of Lipoprotein A is now shown as follows:

Metric	Ancestry group	Predictive performance				n
		Geno	Covars	Full	delta	
R^2	white British (model development)	0.569	0.001	0.190	0.189	205656
R^2	white British (hold-out test set)	0.534	0.001	0.176	0.176	51385
R^2	Non-British white	0.496	0.005	0.049	0.044	19197
R^2	South Asian	0.136	0.008	0.021	0.013	6542
R^2	East Asian	0.057	0.007	0.015	0.007	1452
R^2	African	0.007	0.016	0.017	0.002	5086

The “Predictive performance and transferability in UK Biobank” table for Lipoprotein A in the Global Biobank Engine: https://biobankengine.stanford.edu/RIVAS_HG19/snpnet/INI30790

8. The correlation of number of selected variable and the predictive performance sounds like an issue with power. This is similar to the self-contained test-statistics in gene set studies where including more information

has a higher chance of having a high predictive performance. The lack of correlation in binary traits might be due to rare variants that have large effect or ascertainment of case control. For example, only one variants were selected for Iritis. Also, if we look at the Global Biobank Engine, we can see a lot of duplicated traits that were assigned to different categories. For example, Lipoprotein A is both a biomarker and blood assays. Were this duplicated removed from the correlation analyses? If not, then duplicated traits or highly correlated traits (e.g. Hand grip strength left and right) might have inflated the correlation. Considering the trait definition, it is also much easier to have duplication and correlation between quantitative traits than the binary traits.

Thank you very much for raising this important point.

We totally agree that it is not appropriate to include duplicated traits in the correlation analysis. As we described above (points #3 and #7), there were duplicates in the original version of the manuscript. We had two versions of biomarker traits: one with covariate adjustment and one without adjustment. In the revised version of the manuscript, we removed those duplicates.

The lack of the observed correlation stems from the power limitation in the binary traits or due to the potential differences in the underlying genetic architecture. With the data we have access to, it is not feasible to pinpoint the exact reason. We, therefore, expanded the discussion.

Lines 330-338, Page 13, Discussion

> *When we compared the number of independent loci included in the model and their incremental predictive performance, we found a significant correlation across quantitative traits but not within binary traits. While the underlying genetic architecture of binary traits may span the gamut of a wide variety of polygenicity — from Mendelian and monogenic to oligogenic, omnigenic, and polygenic —, that of highly heritable quantitative traits may not be compatible with monogenic inheritance as illustrated in the wide adoption of Fisher's infinitesimal model[31–33]. In addition to potential differences in the underlying genetic architectures across traits, the limitation in power for some traits, especially for the binary traits with limited case counts, differences in heritability, and a combination of all of those may be the contributing factor to the lack of the observed correlation in binary traits.*

Thank you very much for your suggestion.

Referee #2:

Here the authors investigate properties of PRS across 428 traits in the UK. The work is nicely conducted and explained.

Thank you very much for taking the time to review the manuscript and for providing detailed feedback. We are confident that your comments have improved the clarity of the manuscript. The summary of the key changes in this revision is summarized on the first page of this document. Here are the responses to your suggestions.

The introduction and Discussion need to better sign post what this study IS and what it is NOT about. For example, it is NOT promoting BASIL at the best PRS method, but rather it can be considered as a method that can be easily applied across many traits and could be useful across a range of genetic architectures without explicitly modelling genetic architectures. Also it is NOT proposing the best predictor for a trait because a) it only uses UKB data and not other GWAS data available for some traits b) relatives are excluded which (although independence of discovery and test sample are important) the GWAS discovery could be more powerful by including relatives (I am not saying you need to include the relatives for the purpose of this paper but rather more clearly define its boundaries). The purpose of this study is more about considering the properties of PRS of many traits from the same data set and examining trends across the traits.

Thank you very much for providing specific suggestions on how we can further clarify the scope of the work. Following your advice, we have updated the introduction and discussion sections.

Lines 62-81, Page 3, Introduction

> *Polygenic risk score (PRS), an estimate of an individual's genetic liability to a trait or disease, has been proposed for disease risk prediction with potential clinical relevance for some traits[1,2]. Due to training data sample size increase and methods development advances for variable selection and effect size estimation, PRS predictive performance has improved[3–17]. However, it has not been clear what would be the predictive performance of PRS models when it is applied to a wide range of traits, which may have diverse genetic architecture and their transferability across ancestry groups. Rich phenotypic information in large-scale genotyped cohorts provides an opportunity to address this question.*
>
> *Here, we present significant sparse PRSs across 813 traits in the UK Biobank[18,19]. We applied the recently developed batch screening iterative lasso (BASIL) algorithm implemented in the R snpnet package[10] across more than 1,500 traits consisting of binary outcomes and quantitative traits, including disease outcomes and biomarkers, respectively (Fig. 1, S1 Table). As opposed to most of the recently developed PRS methods that take genome-wide association study (GWAS) summary statistics as input, BASIL/snpnet is capable of performing variable selection and effect size estimation simultaneously from individual-level genotype and phenotype data. BASIL/snpnet results in sparse PRS models, meaning that most genetic variants in the input dataset have zero coefficient. For example, the snpnet PRS for standing height, a classic example of polygenic traits, includes 51,209 variants, which has non-zero coefficients for 4.7% of 1,080,968 genetic variants and allelotypes present in the input genetic data. Moreover, this approach does not require the explicit specification of the underlying genetic architecture of traits, suitable for a phenome-wide application of PRS modeling.*

Lines 340-350, Page 13, Discussion

> *Our study is complementary to many other studies that focus on fewer traits to construct PRS models from GWAS meta-analysis and mixed models. While the sample size in our study is sufficiently large to observe statistical significance in predictive performance across hundreds of traits, it does not necessarily mean the clinical relevance of the PRS models. Moreover, population-based recruitment*

in UK Biobank may not be the best strategy to achieve the highest predictive performance for some traits. A disease-focused study[6,34–36] would be an attractive alternative strategy, especially when multiple genotyped cohorts recruited for the same disease are available or the disease of interest has a low prevalence. Our study, instead, focused on the phenome-wide application of PRS across hundreds of traits in a single cohort by applying BASIL algorithm with readily available implementation in R snpnet package[10], which does not require explicit modeling of underlying genetic architecture across a wide variety of traits.

1. Line 40 Define “sparse PRS”, this may be unclear to some readers.

Thank you very much for your suggestion. Following your advice, we have included the description.

Lines 70-80, Page 3, Introduction

Here, we present significant sparse PRSs across 813 traits in the UK Biobank[18,19]. We applied the recently developed batch screening iterative lasso (BASIL) algorithm implemented in the R snpnet package[10] across more than 1,500 traits consisting of binary outcomes and quantitative traits, including disease outcomes and biomarkers, respectively (Fig. 1, S1 Table). As opposed to most of the recently developed PRS methods that take genome-wide association study (GWAS) summary statistics as input, BASIL/snpnet is capable of performing variable selection and effect size estimation simultaneously from individual-level genotype and phenotype data. BASIL/snpnet results in sparse PRS models, meaning that most genetic variants in the input dataset have zero coefficient. For example, the snpnet PRS for standing height, a classic example of polygenic traits, includes 51,209 variants, which has non-zero coefficients for 4.7% of 1,080,968 genetic variants and allelotypes present in the input genetic data.

2. Lines 55-61 do not define how you made discovery, tuning and testing samples, although the info is in the methods a brief summary is needed to interpret results presented

Thank you very much for your suggestion. We completely agree with you regarding the need for more description of the analysis in the results section so that people would easily interpret the results presented in the manuscript. We expanded our description in the results section.

Lines 116-144, Page 5, Results

Characterizing sparse PRS models with BASIL algorithm

To build sparse PRSs across a wide range of phenotypes, we compiled a total of 1,565 traits in the UK Biobank. We grouped them into trait categories, such as disease outcomes, anthropometry measures, and cancer phenotypes (S1 Table, Methods). We analyzed a total of 1,080,968 genetic variants and allelotypes from the directly-genotyped variants[19], imputed HLA allelotypes[21], and copy number variants[22]. Using 80% (n = 269,704) of unrelated individuals of white British ancestry, we applied batch screening iterative lasso (BASIL) implemented in the R snpnet package[10]. This recently developed method characterizes PRS models by simultaneously performing variable

selection and effect size estimation. Applying different levels of penalization in the Lasso regression with penalty factors, we prioritized the medically relevant alleles in the PRS model. Specifically, we used the predicted consequence of the genotyped variants and the pathogenicity information in the ClinVar database. We prioritized protein-truncating variants, protein-altering variants, imputed HLA allelotype, and known pathogenic and likely-pathogenic variants by assigning lower penalty factors (Methods). As unpenalized covariates, we included age, sex, and the loadings of the top ten principal components (PCs) of genotypes. For 35 blood and urine biomarker traits, we took the snpnet PRS models from a recently published study[23], where the PRS models were characterized with the same methods on the same set of individuals following the adjustment for an extensive list of technical covariates, including fasting time and dilution factors, as well as for age, sex, and genotype PCs.

To evaluate the predictive performance (R^2 for quantitative traits and receiver operating characteristic area under the curve [ROC-AUC] for binary traits) and its significance of PRS models, we focused on the remaining 20 % of unrelated individuals in the hold-out test set (n = 67,425) as well as additional sets of unrelated individuals in the following ancestry groups in UK Biobank: non-British European (non-British white, n = 24,905), African (n = 6,497), South Asian (n = 7,831), and East Asian (n = 1,704) (S2 Table, Methods). We found 813 PRS models with significant ($p < 2.5 \times 10^{-5} = 0.05/2,000$, adjusted for multiple hypothesis testing with Bonferroni method) predictive performance in the hold-out test set of white British individuals (Methods). For the binary traits, we also evaluated pseudo-R^2 using Tjur's Coefficient of Discrimination[24] and Cragg and Uhler's pseudo-R^2 (also commonly known as Nagelkerke's pseudo-R^2)[25,26].

3. Figure 1 Axis labels too small – especially part D- lake plot – not an informative title; The entries of column 1 are not self-evident in terms of discovery/target, from lines 139-145 I see that other ancestries were not included in discovery sample, but not obvious from Fig 1 legend. Line 206, add to avoid ambiguity "The non-British white, African, South Asian and East Asian samples were only used as test sets"

Thank you for providing specific suggestions. We agree with all of your points and updated the figure and the texts.

- We added a new panel (A) in Figure 1 to further clarify the set of individuals used in the study.
- The figure axis labels are now in larger font sizes
- The panel (E) (previously panel D) is now titled "non-zero coefficients of sparse PRS model"
- The ancestry group column in panel (F) (previously panel E) has user-friendly labels
- We added the suggested sentence in the Methods section (now line 426-429, previously line 206).

Lines 426-429, Page 16, Study population and genetic data, Methods

We randomly split the white British cohort into 70% training (n = 235,991), 10% validation (to select the optimal sparsity level) (n = 33,713), and 20% test (n = 67,425) sets[23,57]. We used the same split of training, validation, and test set for all tested traits. The non-British white, African, South Asian, and East Asian samples were only used as test sets.

(A) Study cohort and phenotypes

378,066 unrelated individuals in UK Biobank (**S2 Table**)

337,129 individuals of white British ancestry
- score development (n = 269,704)
- hold-out test set (n = 67,425)

Additional hold-out sets for transferability assessment
- non-British white (n = 24,905)
- African (n = 6,497)
- South Asian (n = 7,831)
- East Asian (n = 1,704)

1,565 phenotypes in UK Biobank (**S1 Table**)

871 quantitative traits

Anthropometry, biomarkers, blood assays, bone densitometry ...

694 binary traits

Disease outcomes, cancer, lifestyle factors, family history ...

Figure 1. Significant sparse polygenic risk scores (PRSs) across 813 traits in the UK Biobank. (A) We analyzed a total of more than 378,000 unrelated individuals and 1,565 traits in UK Biobank. We used 80 % of individuals of white British ancestry for score development. For evaluation, we used the remaining 20 % of individuals and additional individuals in other ancestry groups.

4. I think “ethnic” is now regarded as cultural, and the preferred term in this context is “ancestry”

We agree with your point. Following your suggestion, we replaced “trans-ethnic predictive performance assessment” with “transferability assessment of PRS models across ancestry groups in UK Biobank” in the updated version of the manuscript.

5. Figure 5 would benefit from “quotable” mean number stats for each ancestries

We completely agree with your point. We added the coefficients of a linear regression fit in the main text.

Lines 288-300, Page 11, Results

> ***Sparse PRS models exhibit limited transferability across ancestry groups***
>
> *While the majority of the participants in the UK Biobank are of European ancestry, the inclusion of individuals from African and Asian ancestry enables an assessment of the transferability of the PRS models across ancestry groups in UK Biobank. In addition to the hold-out test set that we derived from the white British population, we focused on additional sets of individuals from non-British European (non-British white), African, South Asian, and East Asian ancestry groups and compared the incremental predictive performance with that in white British hold-out test set (Fig 6). For quantitative traits, the models predicted well for non-British white (linear regression fit of the incremental predictive performance: y = 0.91x), but they suffer limited transferability for the non-European ancestry groups (y = 0.56x, y = 0.47x, and y = 0.13x for South Asian, East Asian, and African, respectively). Similarly, in binary traits, the non-British white showed higher transferability (y =*

> *0.60 x) than the non-European ancestry groups (y = 0.095x, y = 0.011x, and y = -0.086x for South Asian, East Asian, and African, respectively).*

We also included those numbers in figure 6 (previously figure 5) panels as well.

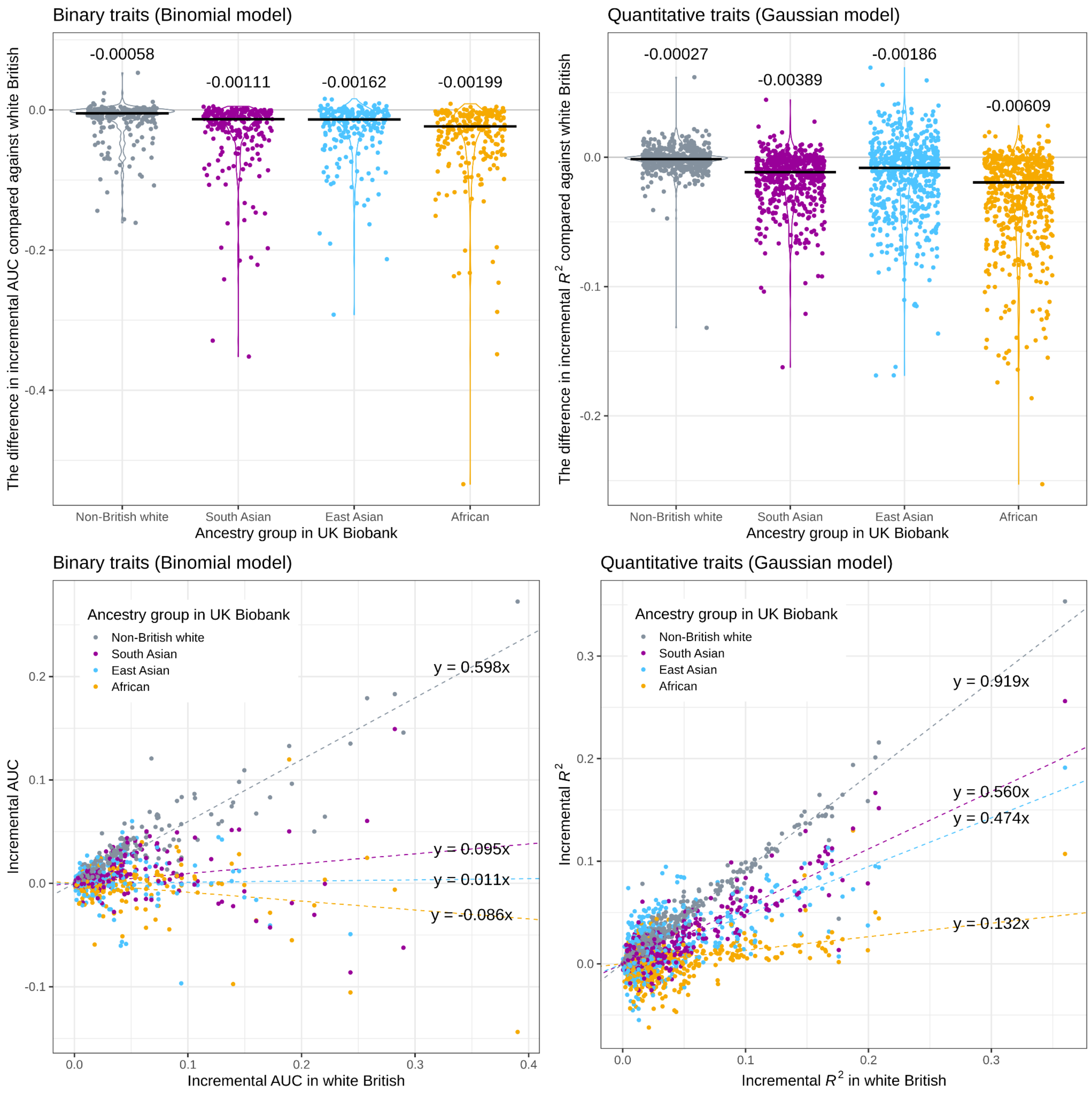

Figure 6. Transferability assessment of PRS models across ancestry groups in the UK Biobank. The incremental predictive performance (incremental AUC for 244 binary traits [left] and incremental R^2 for 569 quantitative traits [right]) was quantified in individuals in different ancestry groups in the UK Biobank and was

compared against that in the hold-out test set constructed from the individuals in white British ancestry group. (Top) the difference in the incremental predictive performance between the target group (x-axis, double-coded with color) and the source white British cohort. The median values are shown as black horizontal bars and numbers. (Bottom) comparison of the incremental predictive performance in the target group (color) and the test set. A simple linear regression fit was shown for each ancestry group with the dashed lines. The slopes of the regression lines were also shown.

6. It would be of interest to have a plot of x-axis SNP-based heritability, y-axis increase in r2/AUC.

Thank you very much for the great suggestion. We added a figure comparing the estimated SNP-based heritability (from LD score regression) and the predictive performance of the PRS model. We used R^2 and Tjur's pseudo-R^2 for quantitative traits and binary traits, respectively.

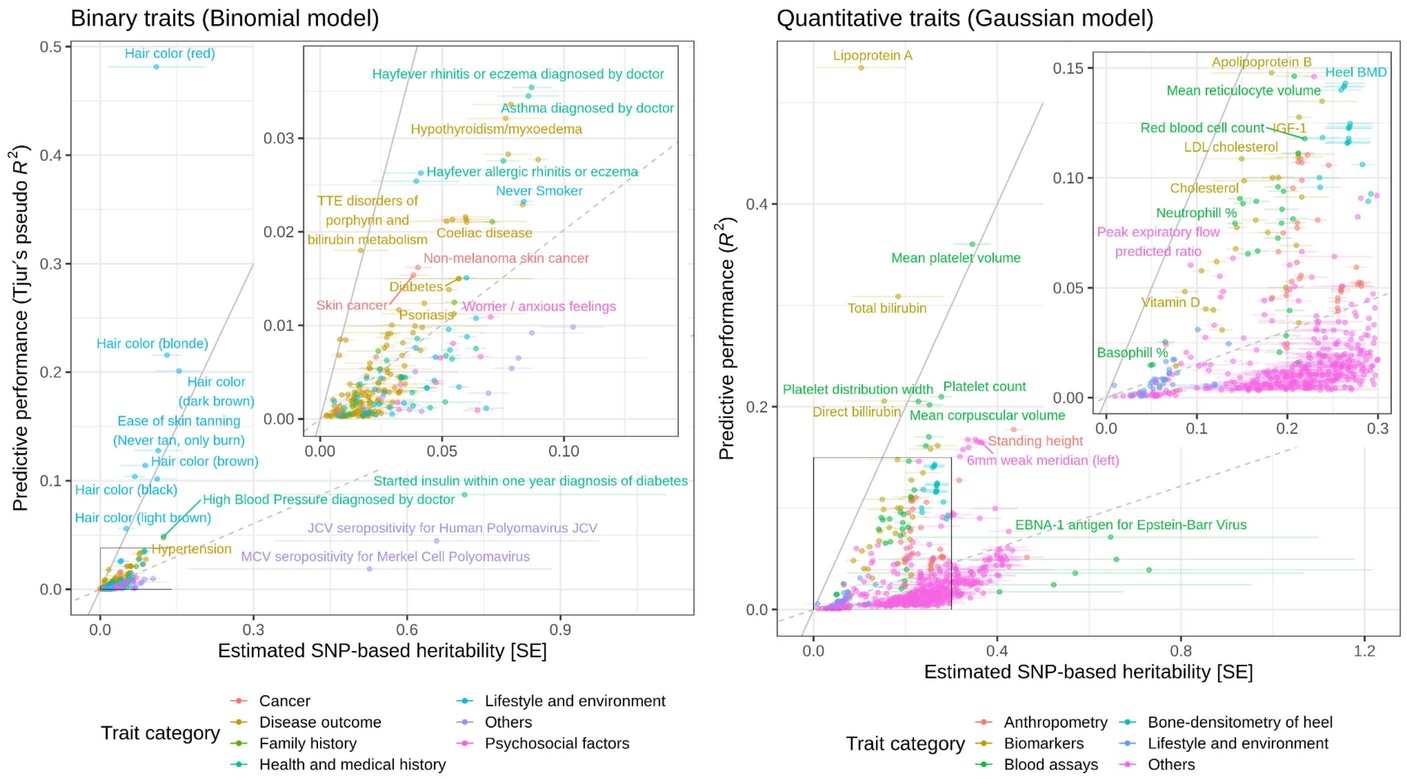

Figure 2. Comparison of the estimated SNP-based heritability and predictive performance across the 813 traits with significant PRSs. The predictive performance (Tjur's pseudo-R^2(ref: [24]) for 244 binary traits [left] and R^2 for 569 quantitative traits [right]) of the PRS models that only consider genetic variants are compared against the estimated SNP-based heritability. The solid gray lines represent y = x, and the dashed gray lines represent a linear regression fit (y = 0.20 x and y = 0.15 x for binary traits and quantitative traits, respectively). We show the points on the bottom left corners in the inset plots. The error bars represent standard error. BMD: Bone mineral density.

7. Given the differing sizes of test sets across ancestries it would be good to remind readers how this does/does not impact on interpretation of cross-ancestry comparisons.

We added a sentence in the discussion section to remind the reader that the non-European ancestry groups in UK Biobank are of a smaller sample size.

Lines 352-361, Page 13, Discussion

> *Like other PRS approaches that consider datasets from one source population in the PRS training, our sparse model trained on the individual-level data of white British showed limited transferability across diverse ancestry groups[37–39]. The sample sizes of non-European ancestry groups in UK Biobank are smaller than that of European ancestry groups. In general, that will result in larger uncertainties in predictive performance assessment. Nonetheless, when we assess the incremental predictive performance across ancestry groups by comparing the full model consisting of the genetic data and basic covariates and the covariate-only model, we found the binary traits, including disease outcomes, have lower transferability compared to quantitative traits, including biomarkers, blood measurements, and anthropometric traits. Improvements of PRS models with high transferability across ancestry groups and the admixed individuals are of interest for future research.*

Referee #3:

In this paper Tanigawa et al. describe the systematic creation of polygenic risk scores (PRS) for > 1,600 traits using data from the UK Biobank (UKB). The construction and evaluation of the PRS is well-described, and the main result of the manuscript is a large resource of PRS built using a single method and a comprehensive web portal describing the results that will be useful for others looking to better understand the performance of each score and apply them to other cohorts. I do not have any major concerns about the manuscript; however, I think some of the unique features of the analysis should be better described and contextualised:

Thank you very much for taking the time to review the manuscript and for providing detailed feedback. We are confident that your comments have improved the clarity of the manuscript. The summary of the key changes in this revision is summarized on the first page of this document. Here are the responses to your suggestions.

• The choice of variants (directly genotyped, imputed HLA, and CNVs) is quite different from classical PRS analyses that usually employs the full-set of imputed variants with MAF/INFO filtering. Does the performance improve if these imputed variants are included in the dataset? It is probably relevant to list the genotyping arrays employed, and adjust for the different arrays used in the performance evaluation.

Thank you very much for asking about the imputed variants and the types of genotyping arrays in UK Biobank.

Following your suggestions, we performed an additional analysis asking whether the inclusion of the imputed variants further improves the predictive performance of the PRS model by focusing on four traits. As you may see below, we did not see a clear difference in predictive performance (for 3 out of 4 traits, the inclusion of the imputed variants helped improve the predictive performance but it was otherwise for the renaming one trait). Nonetheless, we have included this analysis as a supplementary figure. Thank you.

Lines 164-169, Page 6, Characterizing sparse PRS models with BASIL algorithm, Results

> *With the same set of four traits, we asked whether including the imputed genetic variants could improve the predictive performance. We saw some gain in the predictive performance in three traits but not for standing height (S3 Fig). Based on those results, we decided to move on to the phenome-wide application of the BASIL algorithm implemented in the R snpnet packages on the directly genotyped variants, imputed allelotypes, and copy number variants while prioritizing the medically relevant alleles with penalty factors.*

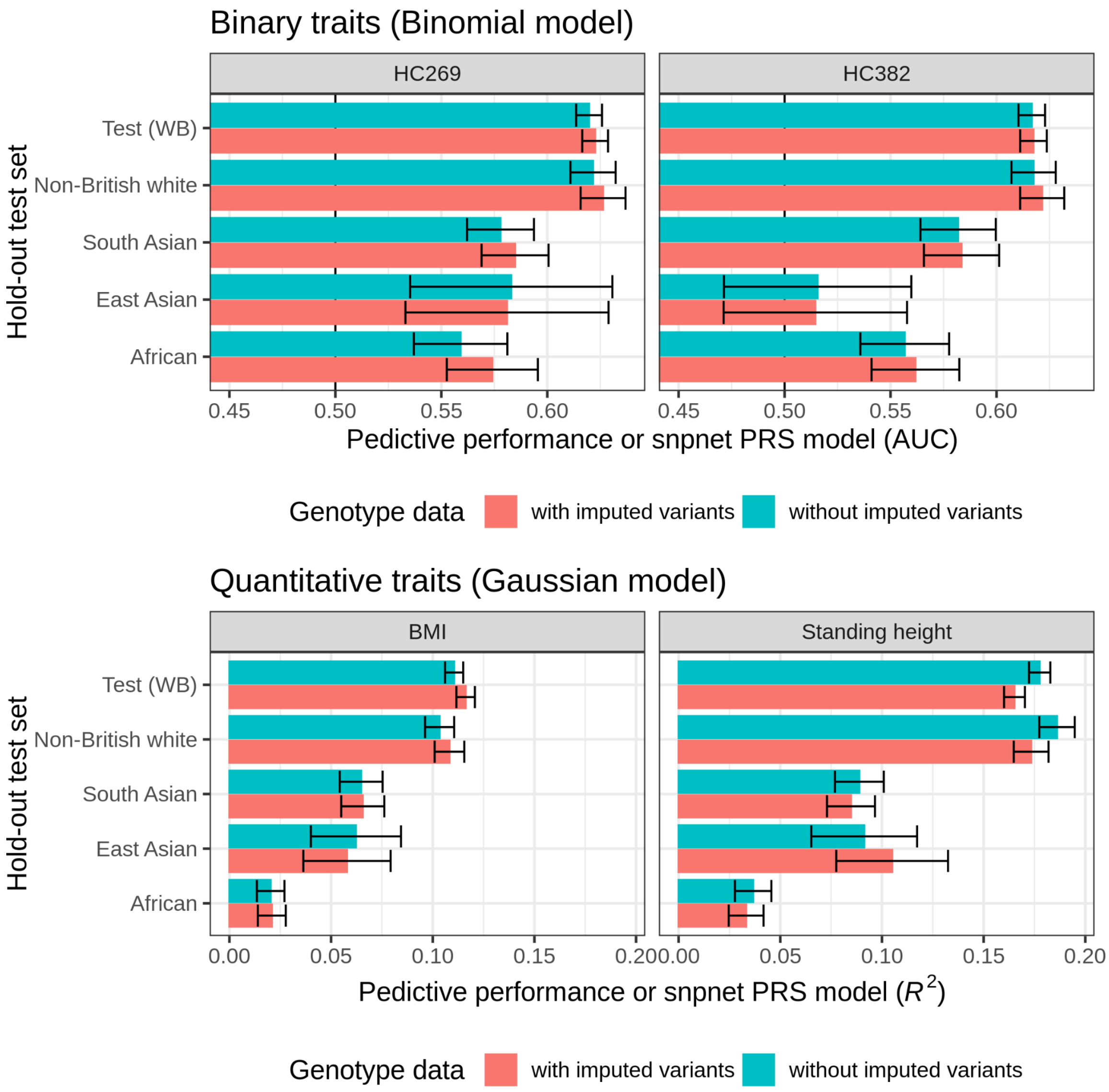

Fig S3. The impact of the imputed genetic variants on the predictive performance of snpnet PRS models. The predictive performance (AUC for binary traits and R^2 for quantitative traits) evaluated across hold-out test set individuals of different ancestry groups in UK Biobank are shown for four traits. The error bars represent the 95% confidence interval.

Regarding the types of genotyping arrays used in the UK biobank, we have revised the performance evaluation of the PRS models and included an indicator variable of the types of arrays in the covariates.

Lines 146-150, Page 5, Characterizing sparse PRS models with BASIL algorithm, Results

> *The participants of the UK Biobank were genotyped on two different arrays: about 10 % of participants were genotyped on the UK BiLEVE Axiom array, whereas the rest were genotyped on the UK Biobank Axiom array[19]. To account for the potential biases correlated with the types of arrays, we evaluated the predictive performance of the PRS by accounting for the types of the arrays in addition to the age, sex, and the top ten genotype PCs.*

Lines 526-547, Page 18, Methods

> ***Predictive performance and transferability of PRS models***
>
> *We evaluated the predictive performance (R^2 for quantitative traits and receiver operating characteristic area under the curve [ROC-AUC] for binary traits) of PRS models. We used the individuals in the hold-out test set (n = 67,425) of white British ancestry as well as additional sets of individuals in non-British white (n = 24,905), African (n = 6,497), South Asian (n = 7,831), and East Asian (n = 1,704) ancestry groups. We evaluated the 95% confidence interval of predictive performance using approximate standard error of R^2 (ref:[63,64]) and DeLong's method[65] for R^2 and AUC, respectively. For the binary traits, we also evaluated pseudo-R^2 using Tjur's Coefficient of Discrimination[24] and Cragg and Uhler's pseudo-R^2 (also commonly known as Nagelkerke's pseudo-R^2)[25,26]. We evaluated the predictive performance of (1) the genotype-only model, (2) the covariate-only model, and (3) the full model that considers both covariates and genotypes. We computed the difference between the full model and the covariate-only model to derive the incremental predictive performance.*
>
> *To evaluate the predictive performance of the covariate-only model in the hold-out test set of white British ancestry, we fit a generalized regression model, trait ~ age + sex + array + Genotype PCs, using the individuals in the score development set. We subsequently computed the risk scores based on the covariate terms for the individuals in the hold-out test set. The array is an indicator variable denoting the types of the genotyping array (either the UK BiLEVE Axiom array or the UK Biobank Axiom array). For the individuals in non-British white, African, South Asian, and East Asian ancestry groups, we took the ancestry group-specific PCs computed for each set[23] and fit the same regression model for each group. We did not use the array indicator variable for African, South Asian, and East Asian because all individuals in those ancestry groups were genotyped on the UK Biobank Axiom Array (S2 Table).*

• The prioritization of medically-relevant (ClinVar pathogenic/likely-pathogenic, VEP predicted protein-truncating/altering variants) for non-zero effect weights in the PRS is also a quite interesting addition; however, I was surprised to see no quantitative analysis of its impact on PRS performance. I would also

hypothesize that the weighting would also impact the number of variants selected in the model (Figure 4)? Some comparison of the PRS performance and transferability with/without the variant prioritisation is necessary.

Thank you very much for your great suggestion. We performed an additional analysis evaluating the effects of the prioritization of the medically relevant alleles focusing on four traits. As you may see below, we found a little difference in the predictive performance in the hold-out test set as well as in other ancestry groups in UK Biobank while we saw an enrichment of the prioritized alleles.

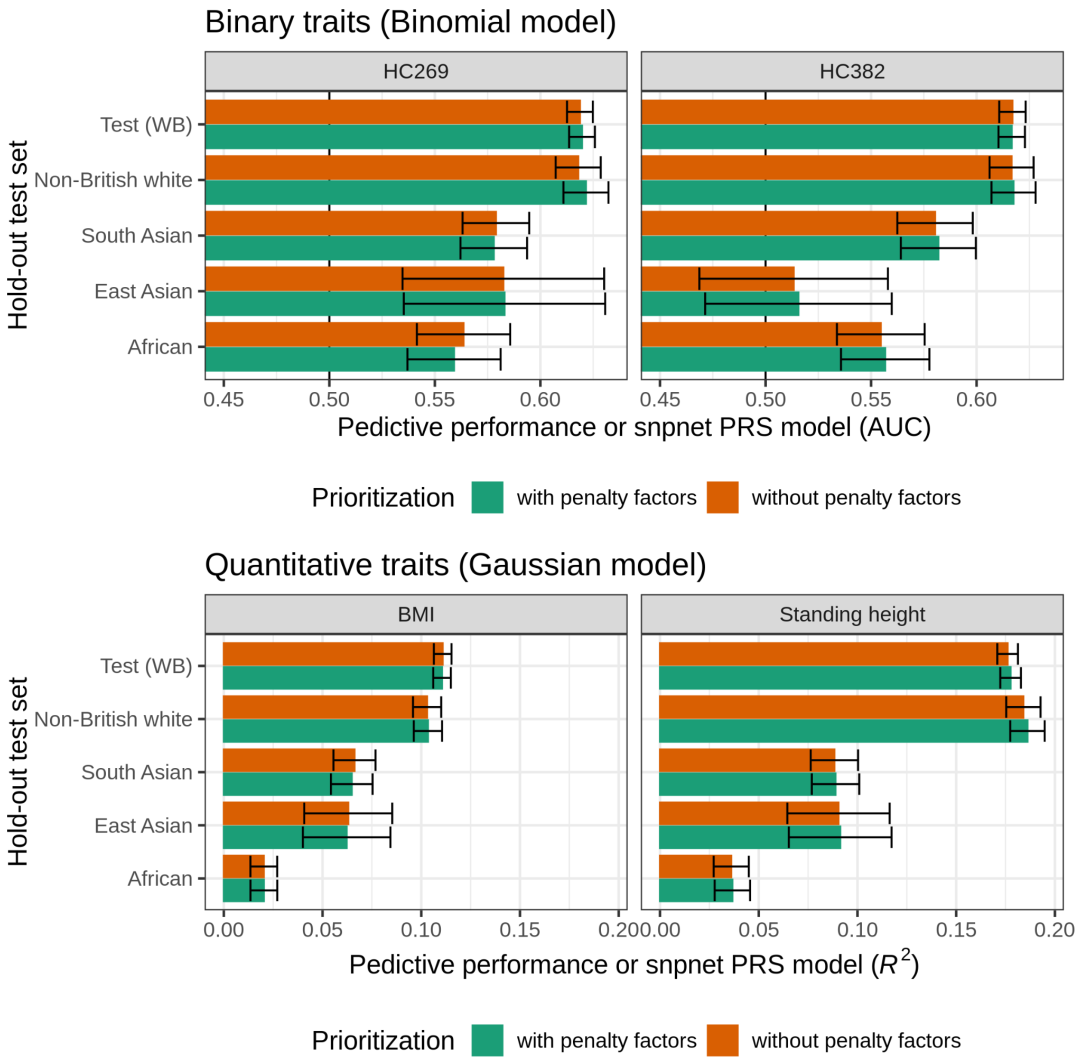

Fig S2. The impact of prioritizing the medically relevant alleles with penalty factors on the predictive performance of snpnet PRS models. The predictive performance (AUC for binary traits and R^2 for quantitative traits) evaluated across hold-out test set individuals of different ancestry groups in UK Biobank are shown for four traits. The error bars represent the 95% confidence interval.

Table 1. The prioritization of the medically relevant alleles with penalty factors. The number of the genetic variants or allelotypes with non-zero coefficient values are shown for the four traits. The denominator

represents the total number of variables included in the model. The numerator represents the number of the medically relevant alleles, which are one of the following: protein-truncating variants, protein-altering variants, imputed HLA allelotypes, the pathogenic or likely-pathogenic variants in the ClinVar database. The enrichment of the medically relevant variants is also shown.

Trait	Number of selected genetic variants or allelotypes		
	with penalty factor	without penalty factor	enrichment
Standing height	4187 / 51209	2129 / 55937	2.15
Body mass index	2543 / 27126	977 / 28667	2.75
High cholesterol	969 / 5987	215 / 5506	4.14
Asthma	1022 / 6430	250 / 6819	4.34

Lines 153-162, Pages 5-6, Results

> *To assess the degree of prioritization of the medically relevant alleles, we selected standing height, body mass index (BMI), high cholesterol, and asthma. We compared the predictive performance and the number of genetic variants for each functional category. For the four selected traits, we found a little difference in the predictive performance (R^2 = 0.177 vs. 0.176 for the PRS model with penalty factor and without penalty factor, respectively, for standing height, R^2 = 0.111 vs. 0.111 for BMI, AUC = 0.620 vs. 0.619 for high cholesterol, and AUC = 0.617 vs. 0.617 for asthma) (S2 Fig) while we saw an enrichment of the number of the medically relevant alleles with non-zero coefficients in the PRS model with prioritization (2.14 fold enrichment standing height, 2.75 fold for BMI, 4.14 fold for high cholesterol, and 4.33 fold asthma) (Table 1, S3 Table), highlighting the flexibility of the BASIL/snpnet in assigning different levels of penalization based on the variant-level information.*

Lines 517-525, Page 18, Construction of sparse PRS models, Methods

> *To prioritize coding variants over non-coding variants in linkage, we assigned three levels of penalty factors (also known as penalty scaling parameter)[62]: 0.5 for pathogenic variants in ClinVar[53] or protein-truncating variants according to VEP-based variant annotation[59]; 0.75 for likely pathogenic variants in ClinVar, VEP-predicted protein-altering variants, or imputed allelotypes; and 1.0 for all other variants. The assignment rules of the penalty factors are summarized in (S5 Table). The variants with lower values of penalty factors are prioritized in the L1 penalized regression. To assess the degree of prioritization of the medically relevant alleles and their impacts on the predictive performance, we focused on four traits (standing height, BMI, high cholesterol, and asthma) and fit a separate model without penalty factors. We compared the number of selected variants and the predictive performance.*

• Are there any obvious reasons that the correlation of predictiveness and number of variants changes? Is it dictated by differences in effect-size distributions or the MAF of selected variants?

Thank you for asking for further clarification of our observation. Indeed, reviewer #1 also asked a similar line of question and suggested the difference in the power between the binary and quantitative traits as one of the possible reasons. As it is not feasible to pinpoint the exact reason for our observation, we expanded the discussion section.

Lines 330-338, Page 13, Discussion

> *When we compared the number of independent loci included in the model and their incremental predictive performance, we found a significant correlation across quantitative traits but not within binary traits. While the underlying genetic architecture of binary traits may span the gamut of a wide variety of polygenicity — from Mendelian and monogenic to oligogenic, omnigenic, and polygenic —, that of highly heritable quantitative traits may not be compatible with monogenic inheritance as illustrated in the wide adoption of Fisher's infinitesimal model[31–33]. In addition to potential differences in the underlying genetic architectures across traits, the limitation in power for some traits, especially for the binary traits with limited case counts, differences in heritability, and a combination of all of those may be the contributing factor to the lack of the observed correlation in binary traits.*

Minor comments:

• A table with the age/sex/follow-up time/ancestry breakdown of the different training and test sets should be included. Were individuals included in the 70% training set consistent across all PRS being built?

Thank you very much for your suggestion. We added Supplementary Table 2 to describe the sample characteristics. We also added panel (A) in figure 1 to further clarify the different sets of individuals used in the manuscript.

(A) Study cohort and phenotypes

378,066 unrelated individuals in UK Biobank (**S2 Table**)
- 337,129 individuals of white British ancestry
  - score development (n = 269,704)
  - hold-out test set (n = 67,425)
- Additional hold-out sets for transferability assessment
  - non-British white (n = 24,905)
  - African (n = 6,497)
  - South Asian (n = 7,831)
  - East Asian (n = 1,704)

1,565 phenotypes in UK Biobank (**S1 Table**)
- 871 quantitative traits
  - Anthropometry, biomarkers, blood assays, bone densitometry ...
- 694 binary traits
  - Disease outcomes, cancer, lifestyle factors, family history ...

Figure 1. Significant sparse polygenic risk scores (PRSs) across 813 traits in the UK Biobank. (A) We analyzed a total of more than 378,000 unrelated individuals and 1,565 traits in UK Biobank. We used 80 % of individuals of white British ancestry for score development. For evaluation, we used the remaining 20 % of individuals and additional individuals in other ancestry groups.

The 70 % training set, the 10 % validation set (to determine the level of sparsity in the penalized regression), and the 20 % test set split is the same across all the PRS models. We clarified these points. We clarified this point in the Methods section.

Lines 495-507, Page 17, Methods

> ***Construction of sparse PRS models***
>
> *Using the batch screening iterative Lasso (BASIL) algorithm implemented in the R snpnet package[10], we constructed the sparse PRS models for the 1,565 traits. We used the Gaussian family and the R^2 metric for quantitative traits, whereas we used the binomial family and the AUC-ROC metric for the binary traits[10]. For each trait, we fit a series of regression models with a varying degree of sparsity on the training set, consisting of 70% (n = 235,991) of unrelated individuals of white British ancestry. The predictive performance of each of the models is evaluated on the validation set, which consists of 10% (n = 33,713) of unrelated individuals of white British ancestry to guide the selection of the optional level of sparsity. We selected the sparsity that maximizes the predictive performance in the validation set. We subsequently refit the penalized regression model using the individuals in the combined training and validation set individuals (n = 269,704), which we denote as score development set, to maximize the power in the regression model[10]. We used the same training, validation, and test set split across all the PRS models analyzed in this study.*

• Description of how the p-value threshold for incremental predictiveness was selected should be provided.

Thank you very much for your suggestion. We clarified the p-value threshold in the results and the methods section.

Lines 135-142, Page 5, Characterizing sparse PRS models with BASIL algorithm, Results

> *To evaluate the predictive performance (R^2 for quantitative traits and receiver operating characteristic area under the curve [ROC-AUC] for binary traits) and its significance of PRS models, we focused on the remaining 20 % of unrelated individuals in the hold-out test set (n = 67,425) as well as additional sets of unrelated individuals in the following ancestry groups in UK Biobank: non-British European (non-British white, n = 24,905), African (n = 6,497), South Asian (n = 7,831), and East Asian (n = 1,704) (S2 Table, Methods). We found 813 PRS models with significant ($p < 2.5 \times 10^{-5} = 0.05/2,000$, adjusted for multiple hypothesis testing with Bonferroni method) predictive performance in the hold-out test set of white British individuals (Methods).*

Lines 554-557, Page 19, Predictive performance and transferability of PRS models, Methods

> *We looked at the p-value reported for the PRS term for the statistical significance of the PRS model. We used $p < 2.5 \times 10^{-5}$ (= 0.05/2000, adjusted for multiple hypothesis testing using the Bonferroni method for the number of traits analyzed in the study) as the significance threshold.*

• A major advantage of the BASIL/snpnet application in comparison to other PRS-derivation methods seems to be that it does not rely on LD reference panels which often limit the PRS derivation set to being a

single-ancestry group. Given that the manuscript is somewhat focused on transferability of sparse PRS: would it be possible to derive new PRS using a random sample of the entire cohort (all ancestries) and evaluate how the multi-ancestry PRS compare to European-PRS at the whole population and single-ancestry level? [I realize this is beyond the scope of the current analysis but would be informative and may greatly improve the impact]

Thank you very much for pointing out the requirement of individual-level data as one of the unique features of the BASIL/snpnet. We agree with your insightful comments but such investigation is beyond the scope of the current work. We updated the discussion section.

Lines 352-361, Page 13, Discussion

> *Like other PRS approaches that consider datasets from one source population in the PRS training, our sparse model trained on the individual-level data of white British showed limited transferability across diverse ancestry groups[37–39]. The sample sizes of non-European ancestry groups in UK Biobank are smaller than that of European ancestry groups. In general, that will result in larger uncertainties in predictive performance assessment. Nonetheless, when we assess the incremental predictive performance across ancestry groups by comparing the full model consisting of the genetic data and basic covariates and the covariate-only model, we found the binary traits, including disease outcomes, have lower transferability compared to quantitative traits, including biomarkers, blood measurements, and anthropometric traits. Improvements of PRS models with high transferability across ancestry groups and the admixed individuals are of interest for future research.*
