# Supplementary material for: Significant sparse polygenic risk scores across 813 traits in UK Biobank
Source: PLoS Genet. 2022 Mar 24;18(3):e1010105. doi: 10.1371/journal.pgen.1010105 (PMC8946745; doi:10.1371/journal.pgen.1010105)
Supplement: Attachment — Submitted filename: 2022.01.27 10.34 PRSmap response to reviewers (2022 Jan).pdf [file pgen.1010105.s012.pdf]

Please find the review response and revision regarding our manuscript “Significant Sparse Polygenic Risk Scores across 813 traits in UK Biobank” (PGENETICS-D-21-01210R1). Our responses to the reviewers’ individual comments below are in blue font, the comments from the reviewer are copied in black, and quoted texts from the updated manuscript are shown in gray with a vertical bar (examples are shown below):

This is an example of the reviewer’s comments
This is an example of our response.

> *This is an example of quoted texts from the updated manuscript*

We revised the manuscript based on the feedback from the reviewers. The major points of the revision include:

- Given the feedback from reviewers #1 and #2, we removed the sentences that inappropriately mentioned genetic architecture in binary traits. We instead clarified that there is a power difference between quantitative traits and binary traits.
- Given the concerns (from reviewer #2) regarding the lack of theoretical basis in using incremental ROC-AUC for assessing linear relationship (estimated SNP-based heritabilities and transferability assessment), we now use Nagelkerke's pseudo-R^2 as the primary evaluation metric of predictive performance for binary traits in the current version of the manuscript.
- As we change the evaluation metric for binary traits, we now observe a significant rank-based correlation between the effect size (incremental Nagelkerke's pseudo-R^2) and the model size (number of genetic variants with non-zero coefficients) of the sparse PRS model.

Our specific point-to-point responses to the reviewer’s comments are listed below.

Reviewer #1:

This manuscript has certainly been improved with the addition of more detail descriptions of the method and procedure involved. Thank you to the authors for making all these efforts.

Overall, I am still slightly confused as to what are the main messages of the current paper. I am also slightly concern about some interpretation of the results.

Thank you very much for taking the time to review the manuscript and for providing detailed feedback. We are confident that your comments have improved the clarity of the manuscript. Here are the responses to your suggestions.

1. While the authors have now included much of the needed details regarding the procedure and methods performed, there are still some critical information that are missing. For example, quantitative traits were calculated as the "median of non-NA values, as described elsewhere", does that mean that the authors took the measurement across multiple assessment time points and took the median of that? Did the author perform any quality controls on the phenotype to remove outliers?

Thank you very much for clarifying the phenotype definition. Indeed, some of the phenotypes are collected at multiple time points (called "instances" in UK Biobank) at the assessment center. The non-NA median was taken across those multiple timepoints. We have clarified this in the updated texts in the manuscript.

Lines 470-479, Page 17, Methods

> ***Phenotype definitions in the UK Biobank***
>
> *We analyzed a wide variety of traits in the UK Biobank, including disease outcome[46,60], family history [46,60], cancer registry data[46], blood and urine biomarkers[23], hematological measurements, and other binary and quantitative phenotypes[55,56]. Some phenotype information collected at UK Biobank's assessment center contains up to four instances, each of which corresponds to (1) the initial assessment visit (2006-2010), (2) first repeat assessment visit (2012-2013), and (3) imaging visit (2014-), and (4) first repeat imaging visit (2019-). Briefly, for binary traits, we performed manual curation of phenotypic definitions and assigned "case" status if the participants are classified as case in at least one of their visits and "control" otherwise. For quantitative traits, we took the median of non-NA values, as described elsewhere[55].*

The phenotypes analyzed in the present study were derived using the procedure described in the previously published studies. As we did not derive any new phenotype, we did not perform additional phenotype quality controls in this study. As you will see in the response to your log-transformation of the biomarker phenotypes, outlier values of the biomarker measurements were flagged by UK Biobank and we have removed such outliers in our analysis.

2. In a similar vein, for the blood and urine biomarkers, the covariate adjusted phenotype were calculated using the log transformed phenotypic value and the incremental predictive performance were calculated against the predictive value based on the original measurement. Were the original measurements also log transformed? Or was the untransformed value being used? If it is the latter, wouldn't that introduce some bias? In addition, it

is not uncommon to have blood or urine biomarker measurement of 0. In those scenarios, log transformation will lead to undefined value. How was that accounted for?

Thank you very much for the clarification. The original values were not log-transformed with the exception of the three derived traits (eGFR, AST/ALT ratio, and non-albumin protein, where the “original” phenotype values were not available as those values are derived from other biomarker values).

The UK Biobank scientists performed phenotype quality control for the biomarker values (https://biobank.ndph.ox.ac.uk/showcase/showcase/docs/serum_biochemistry.pdf). We excluded the “Outside of the Observed Reportable Range” and QC failed measurements from our analysis, as described in our previously published paper (PMID: 33462484). After removing those measurements, we did not observe zero value in the biomarker measurement value. As such, we simply took the log transformation.

To investigate whether the inclusion of the log-transformed traits introduces unexpected biases, we performed sensitivity analysis where we excluded all of the biomarker traits and repeated all the analyses. The correlation between the estimated SNP-based heritability and predictive performance of the PRS models (R^2), distribution of incremental predictive performance, the correlation between the size of the PRS model (the number of variants with non-zero BETA value) and predictive performance of the PRS models (R^2), as well as the transferability assessment within UK Biobank populations were all largely consistent with the original analysis.

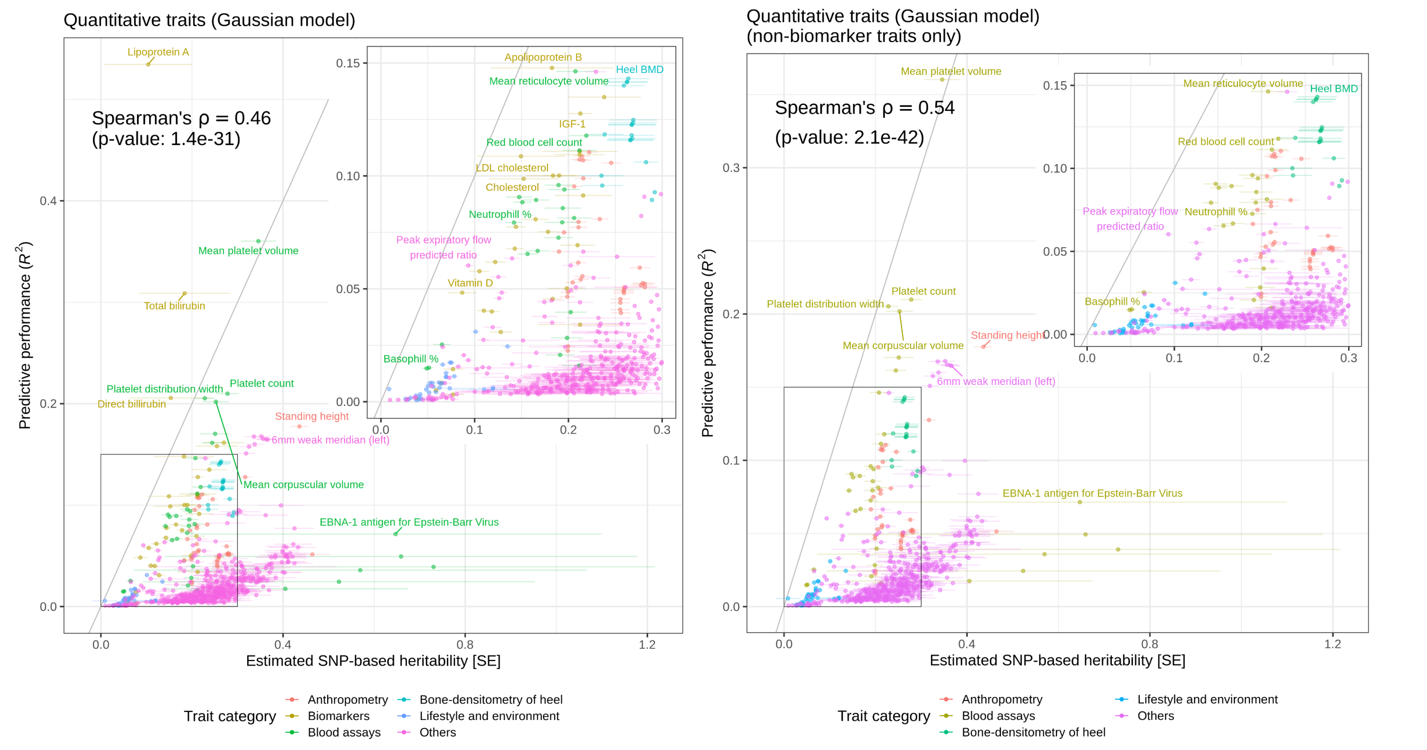

Figure. Comparison of the estimated SNP-based heritability and predictive performance. The

predictive performance (R^2) of the PRS models that only consider genetic variants are compared against the estimated SNP-based heritability. The plots are shown for all 569 quantitative traits (left) and 535 non-biomarker traits (right). The solid gray lines represent y = x. We show the points on the bottom left corners in the inset plots. The error bars represent standard error. BMD: Bone mineral density.

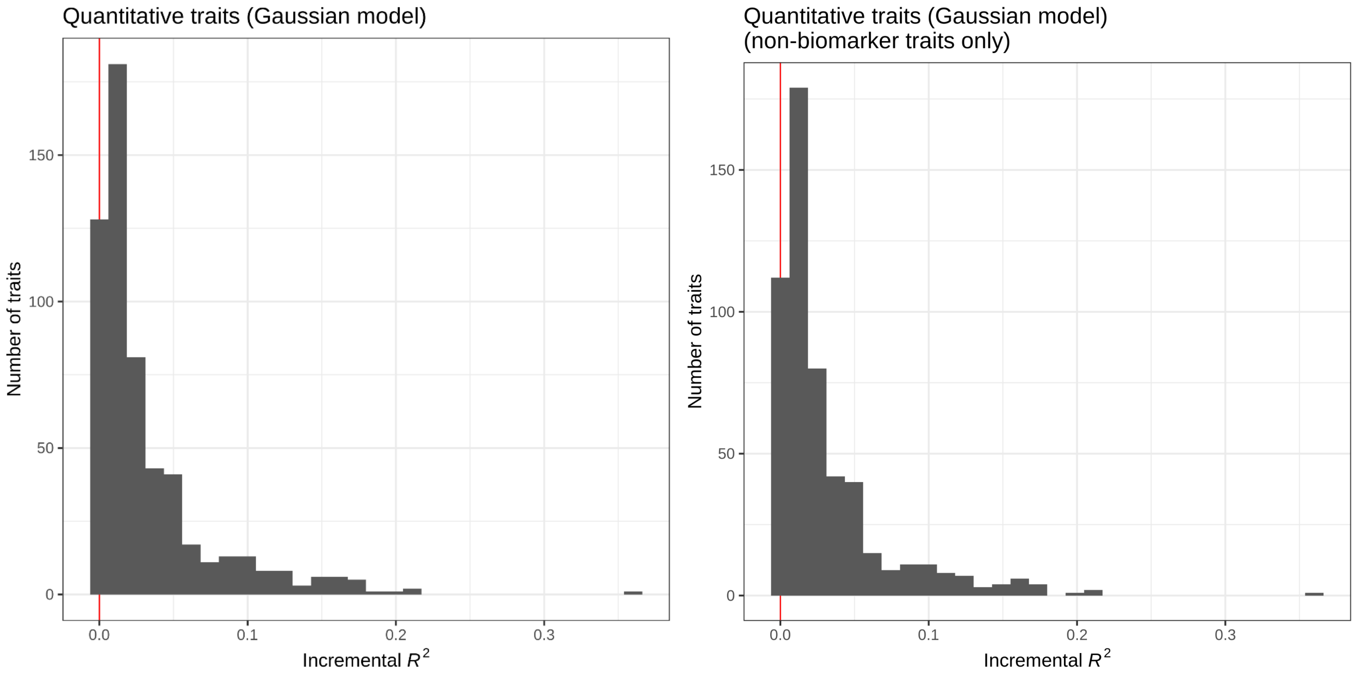

Figure. Incremental predictive performance of the PRS model across the quantitative traits with significant predictive performance in the hold-out test set individuals of white British ancestry. The predictive performance (R^2) of the full models that consider both the genotype and covariates are compared against that of the covariate-only models, and their difference (the incremental predictive performance) is shown as a histogram. The plots are shown for all 569 quantitative traits (left) and 535 non-biomarker traits (right).

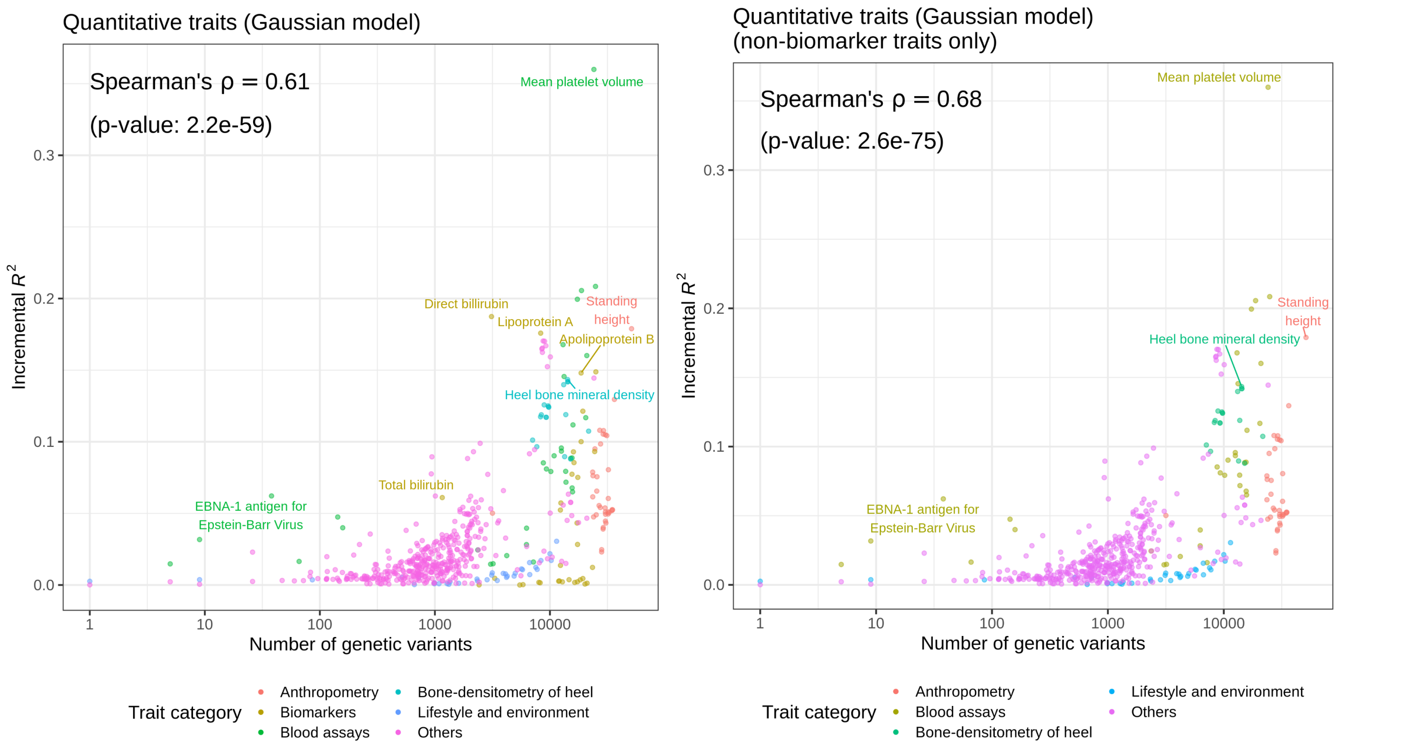

Figure. Comparison of the effect size and the model size of sparse PRS. The number of the genetic variants included in the model (size of the model, x-axis) and the incremental predictive performance (effect size of the model, y-axis) are shown. The plots are shown for all 569 quantitative traits (left) and 535 non-biomarker traits (right).

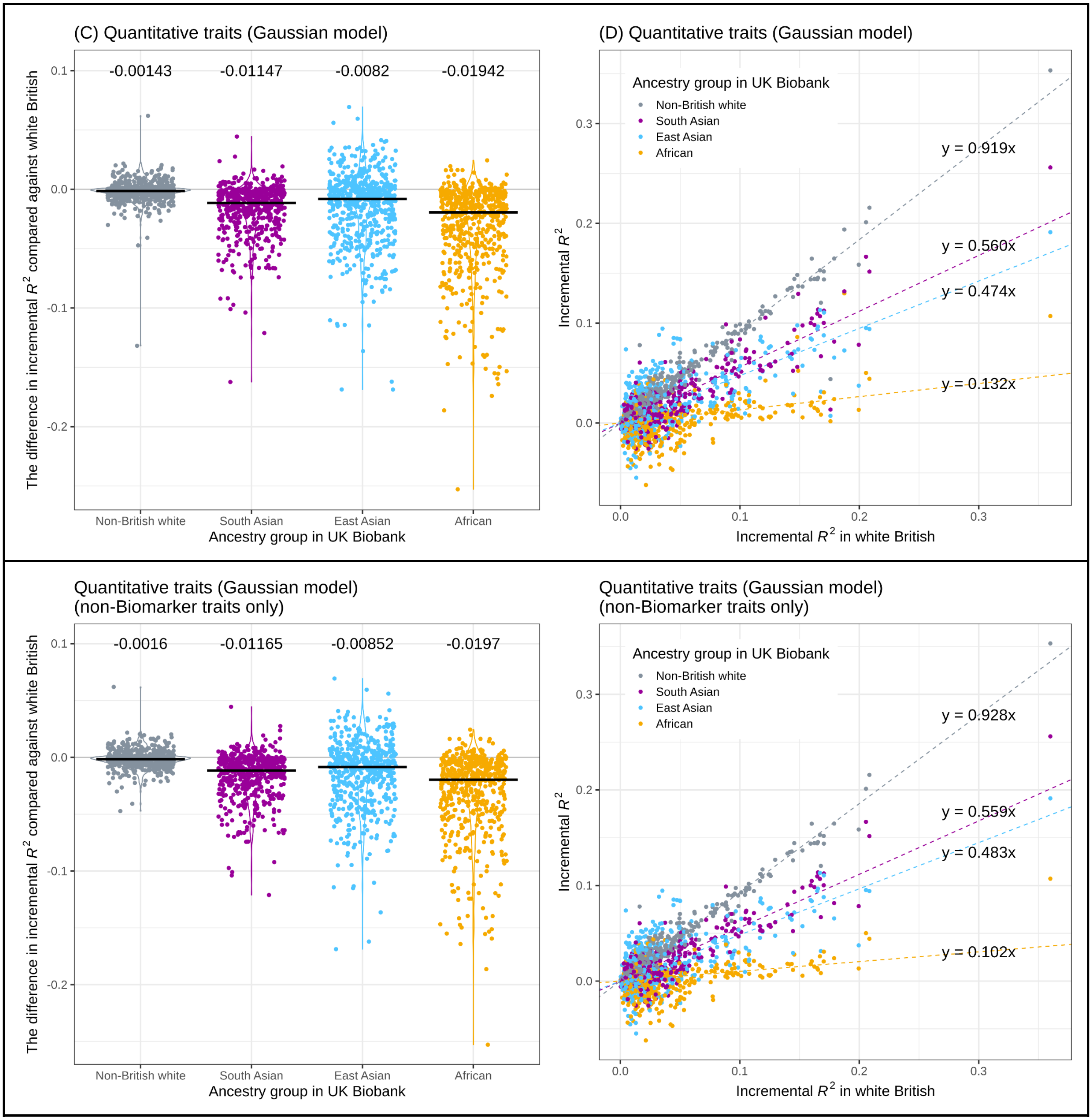

Figure. Transferability assessment of PRS models across ancestry groups in the UK Biobank. The incremental predictive performance (incremental R^2) was quantified in individuals in different ancestry groups in the UK Biobank and was compared against that in the hold-out test set constructed from the individuals of white British ancestry. The plots are shown for all 569 quantitative traits (top) and 535 non-biomarker traits (bottom). (Left) the difference in the incremental predictive performance between the target group (x-axis, double-coded with color) and the source white British cohort. The median values are shown as black horizontal bars and numbers. (Right) comparison of the incremental predictive performance in the target group (color) and the test set. A simple linear regression fit was shown for each ancestry group with the dashed

lines. The slopes of the regression lines were also shown.

3. For the SNP-heritability estimates, the authors perform GWAS on the quantile normalized phenotype. Were the phenotypes also log transformed? It is difficult to assess the relationship between the PRS performance and SNP-heritability if they were performed on phenotypes undergone different transformation. Also, was the quantile normalization done on both quantitative traits and binary traits?

Thank you very much for your clarification question. The quantile normalization was performed only for the quantitative traits when performing the GWAS analysis. We did not apply the quantile normalization in the PRS analysis. This is clarified in the current version of the manuscript.

Lines 571-573, Page 19, SNP-based Heritability estimation, Methods

> *In the regression analysis, we standardized the variance of the covariates (--covar-variance-standardize option) and applied quantile normalization for the quantitative phenotype (--pheno-quantile-normalize option). Note, we did not perform quantile normalization in the PRS analysis.*

In the previous version of Figure 2 (a plot comparing estimated SNP-based heritability and predictive performance), we had a linear regression fit summarizing the relationship between the two variables. We agree with your concern and dropped the linear regression fit. Instead, we report Spearman’s rank-based correlation to indicate the relationship between the estimated heritability and predictive performance without assuming the linear relationship between the two.

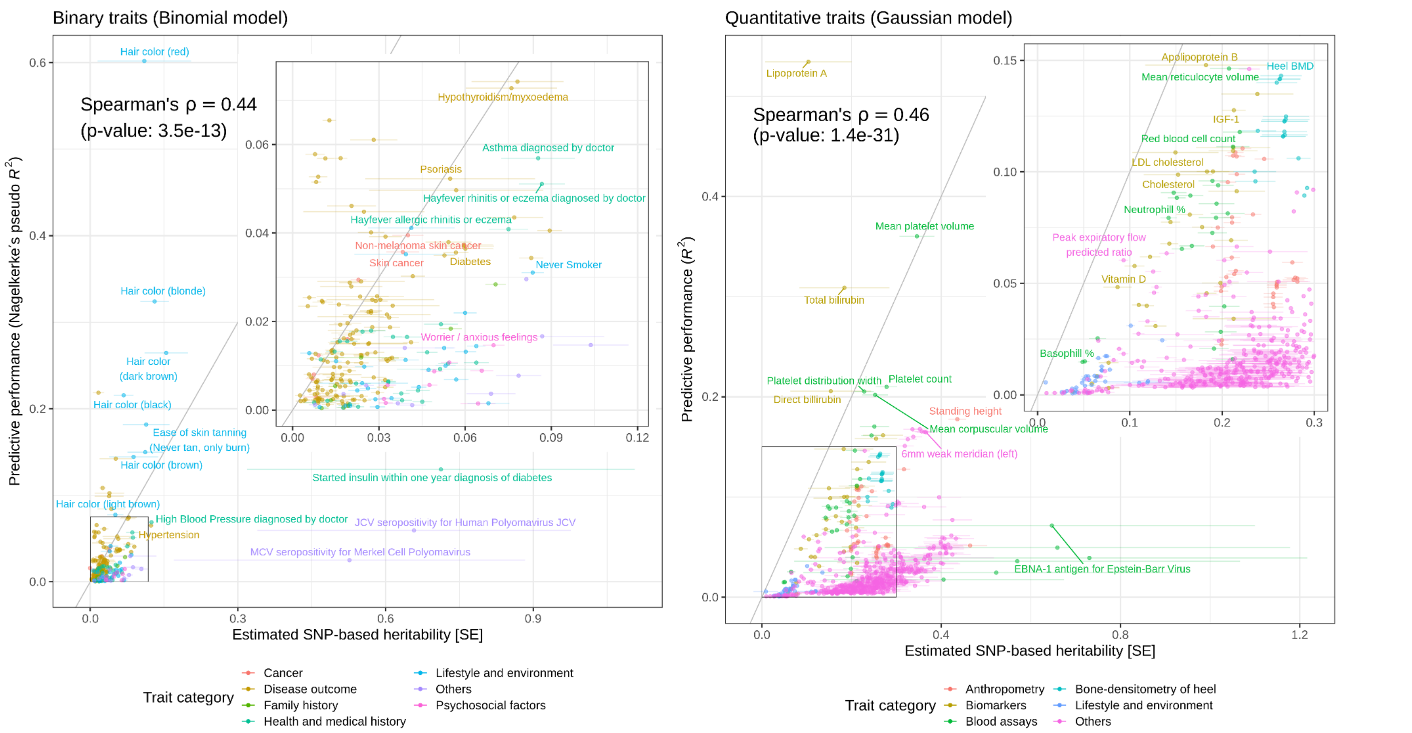

Figure 2. Comparison of the estimated SNP-based heritability and predictive performance across the 813 traits with significant PRSs. The predictive performance (Nagelkerke's pseudo-R^2 for 244 binary traits [left] and R^2 for 569 quantitative traits [right]) of the PRS models that only consider genetic variants are compared against the estimated SNP-based heritability. The solid gray lines represent y = x. We show the points on the bottom left corners in the inset plots. The error bars represent standard error. BMD: Bone mineral density.

4. It is odd to have PRS that report a higher predictive performance than SNP-heritability, as the SNP-heritability are the theoretical upper bound of the PRS. It will be helpful if the authors can provide an explanation as to why the PRS performance is higher than the SNP-heritability (possibly due to different phenotypic transformation, or that the PRS include information that were excluded from the SNP-heritability estimate?). Standard error of the predictions should ideally be also reported to provide a better understanding of the power.

Thank you very much for clarifying the consistency of the reported predictive performance and the estimated heritability. For most traits analyzed in the study, we observed the predictive performance of the PRS models was lower than the SNP-based heritability estimates. The exceptions include hair color traits, lipoprotein A, and total bilirubin. We think the heritability estimates for those traits have downward biases due to the presence of the strong-acting alleles in a few loci.

We used LD score regression (LDSC, PMID: 26414678) to estimate the SNP-based heritability. LDSC estimates the SNP-based heritability of the trait by fitting a linear regression model for the chi-squared statistics on the LD score, which summarizes the degree of linkage with neighboring genetic variants. LDSC removes GWAS associations with extreme association statistics (discussed in the

software's GitHub page: https://github.com/bulik/ldsc/issues/144), potentially introducing downward bias in the heritability estimates.

Taking "hair color (red)", "hair color (blonde)", "hair color (dark brown)", "Lipoprotein A", and "Total bilirubin" as example traits where we have higher predictive performance than estimated SNP-based heritability from LDSC, we examined the GWAS Manhattan plots. As you will see in the plots below, those traits all have extremely large chi-squared association statistics (as well as -log10(P) value).

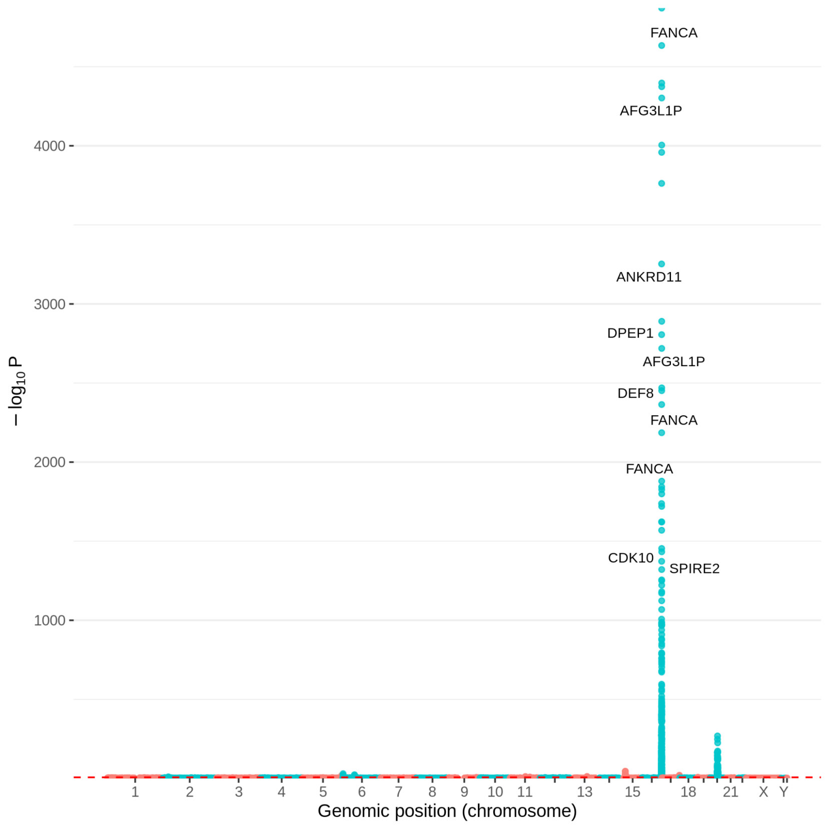

Figure Manhattan plot for Hair color (natural, before graying) red.

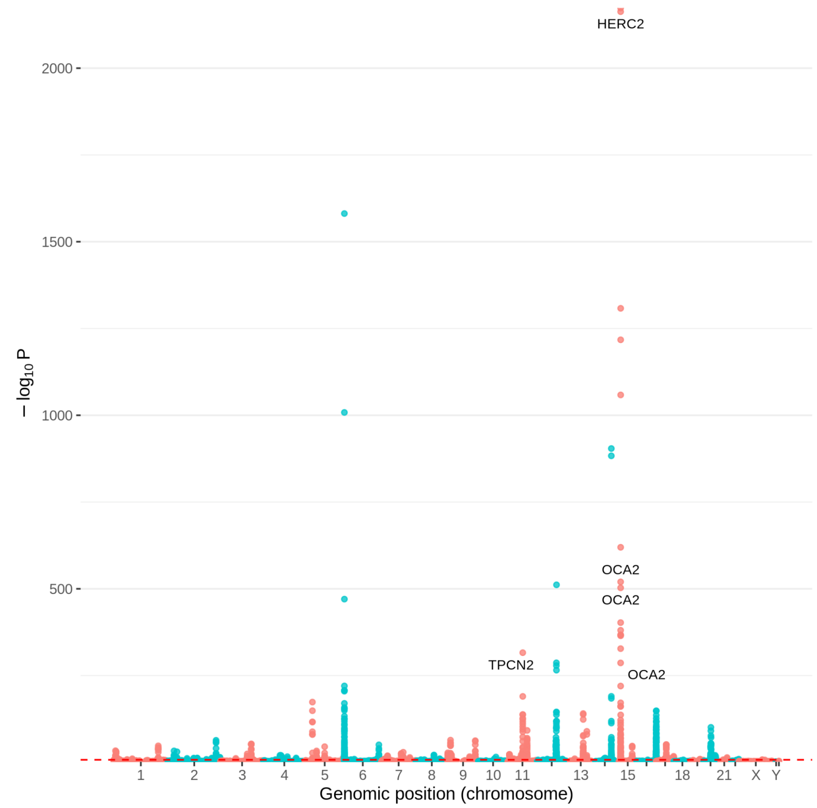

Figure Manhattan plot for Hair color (natural, before graying) blonde.

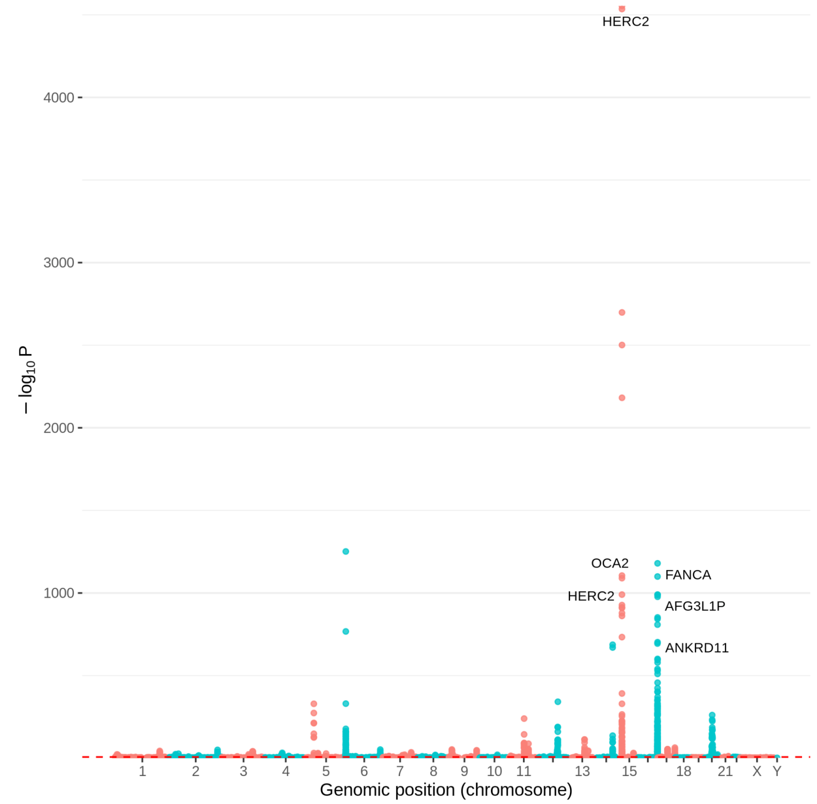

Figure Manhattan plot for Hair color (natural, before graying) dark brown.

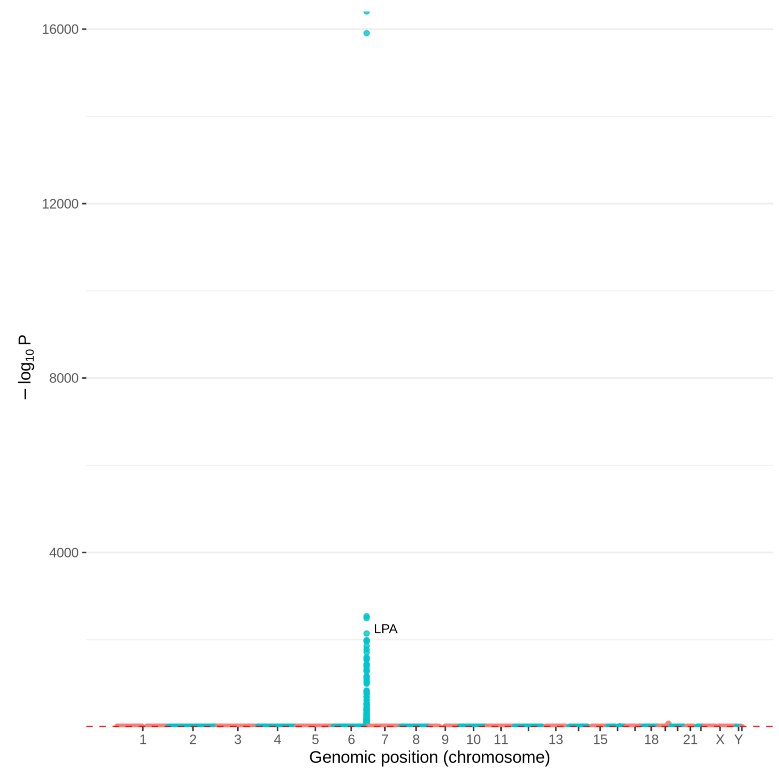

Figure Manhattan plot for Lipoprotein A.

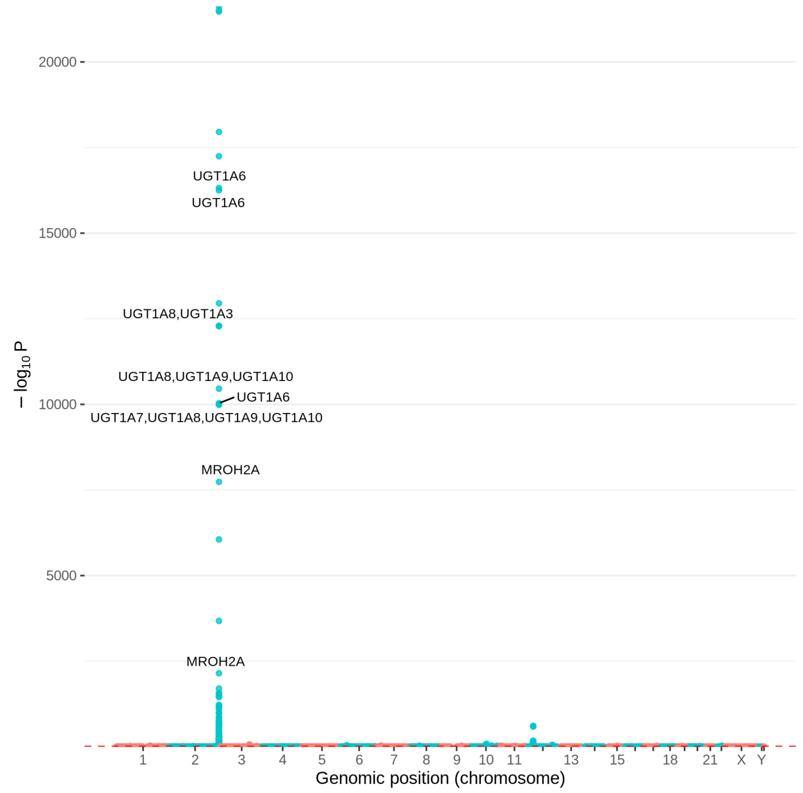

Figure Manhattan plot for Total bilirubin.

When we check the LD score intercept of the GWAS summary statistics of those traits, their LD score intercept values are smaller than that of body mass index (BMI).

Table. LD score regression-based heritability estimates and LD score intercept for selected traits. The full table is available as Supplementary Table S7.

Trait	h2_obs	h2_obs_se	intercept	intercept_se
Hair color (natural, before graying) red	0.1097	0.0949	1.014	0.0111
Hair color (natural, before graying) blonde	0.1307	0.029	1.0556	0.0125
Hair color (natural, before graying) dark brown	0.1542	0.044	1.0538	0.0119
Lipoprotein A	0.1038	0.0964	1.0151	0.0109
Total bilirubin	0.1841	0.1006	1.0364	0.0114
Body Mass Index (BMI)	0.2221	0.0096	1.0671	0.0116

Nonetheless, we agree that it is not appropriate to emphasize that we observe higher predictive performance than SNP-heritability in the main text. We dropped the sentence.

Lines 199-204, Page 7, Results

> *Across 244 binary traits and 569 quantitative traits with significant PRS models, we found higher estimated observed-scale heritability for quantitative traits. Overall, we found a significant correlation between the estimated SNP-based observed-scale heritability and predictive performance (Spearman's rank correlation coefficient ρ = 0.44, p-value = 3.5 x 10-13 for binary traits, ρ = 0.46, p-value = 1.4 x 10-31 for quantitative traits).*

5. Based on how this paper is structured, it seems like the main message is that there is a significant positive correlation between the number of active variables in the PRS model and the incremental predictive performance in quantitative traits but not in binary traits, and this "highlighting the presence of diverse genetic architecture across disease outcomes.". However, because the population prevalence of the binary traits is usually not known, and that the UK Biobank is a prospective cohort where the case numbers might not reflect the true population prevalence, the prediction performance of the binary traits, and their SNP-heritability estimations will likely be biased by ascertainment. In addition, in the main analysis, the authors "used the same split of training, validation and test set for all tested traits.", which means that the case control ratio for the binary traits are likely different between the different set of samples, leading to a greater disparity of performance. Considering the lower heritability of binary traits (mean = 0.04 for binary trait, mean = 0.23 for quantitative traits, based on provided supplementary), reporting on observed instead of liability scale, and the different level of ascertainment bias, it is not surprising that the correlation between the number of active

variables in the PRS model and the incremental predictive performance in binary traits are not significant. And it might be slightly misleading to conclude that the lack of correlation in binary traits, but in quantitative traits is a result of “the presence of diverse genetic architecture”.

Thank you very much for raising important concerns about our previous statements on the interpretation of the results. Based on your feedback and comments from reviewer #2, we (1) dropped the inappropriate sentences mentioning the “diverse genetic architecture”, (2) swapped incremental ROC-AUC with Nagelkerke's pseudo-R^2, and (3) found that there was a significant correlation between the number of active variables and the incremental predictive performance for both quantitative and binary traits. We also acknowledged the presence of the power difference between binary traits and quantitative traits as well as the difference in the observed-scale trait heritability.

Lines 269-274, Page 10, Results

> *We examined whether there is a relationship between the number of active variables in the significant PRS model and the incremental predictive performance. The significant correlation between the two quantities is stronger in quantitative (Spearman’s rank correlation coefficient $\rho = 0.61$, $p = 2.2 \times 10^{-59}$) traits than in binary ($\rho = 0.21$, $p = 9.6 \times 10^{-4}$), reflecting the difference in power between binary and quantitative traits[31].*

Lines 322-327, Page 13, Discussion

> *We assessed the effect size of the PRS model by quantifying the incremental predictive performance, which we define as the difference in the predictive performance between the covariate-only model and the full model consisting of both covariates and genetics. In both quantitative and binary traits, we find a significant correlation between the number of independent loci included in the model and their incremental predictive performance.*

Lines 350-356, Page 13, Discussion

> *Nonetheless, when we assess the incremental predictive performance across ancestry groups by comparing the full model consisting of the genetic data and basic covariates and the covariate-only model, we found the binary traits, including disease outcomes, have lower transferability compared to quantitative traits, including biomarkers, blood measurements, and anthropometric traits. The power difference between binary and quantitative traits[31], limitation in power for some traits, especially for the binary traits with limited case counts, and differences in heritability may be the contributing factors of the observed difference.*

Also, as you correctly pointed out, we used the same set of training, validation, and test set split across all the samples. Those sets are all derived from white British individuals in UK Biobank and

they are equally affected by the ascertainment bias in the population-based cohort. Nonetheless, to investigate your concerns of potential difference in the case frequency in the score development set (training and validation set) and the evaluation set (test set). As you can see in the plot below, we did not see notable differences between the two across 244 binary traits (including disease outcomes and non-disease traits) with significant PRS.

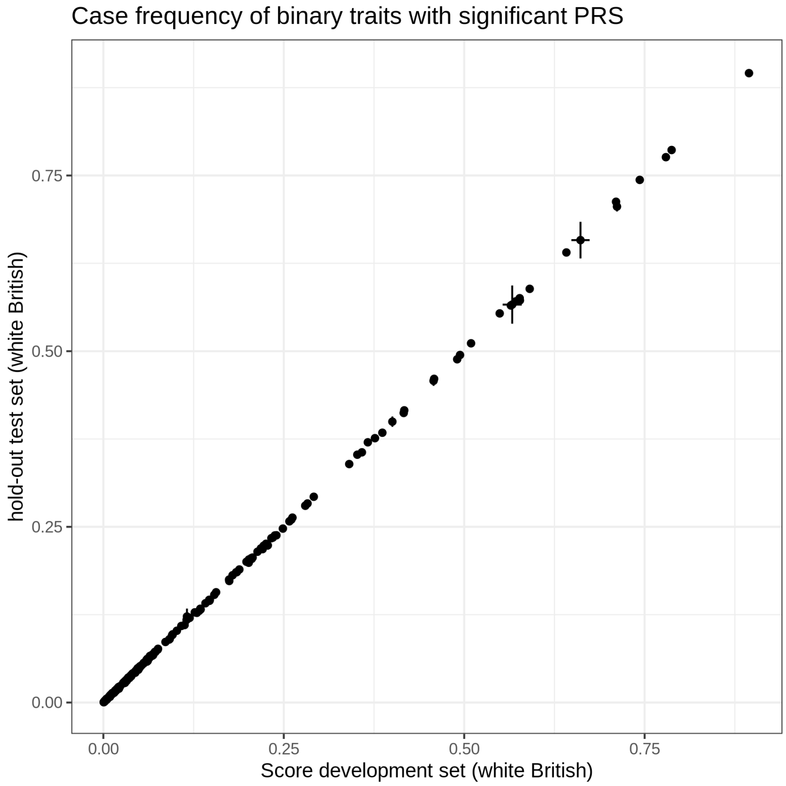

Figure. The case frequency difference between the score development set (training set and validation set, x-axis) and the hold-out test set (test set, y-axis). The error bars represent 95% confidence intervals.

6. Similar to the above comment, the case control ratio in different population might also differ, which was not accounted for here.

We refit the logistic regression across additional populations when evaluating the predictive performance. The intercept term in the logistic regression accounts for the difference in the case prevalence. This is now clarified in the text.

Lines 553-556, Page 19, Methods

> *To evaluate the predictive performance of the full model, we fit a model, trait ~ 1 + covariate-only score + PRS, using the covariate-only score and PRS described above. The constant term accounts for the potential differences in the trait mean (for quantitative traits) or case prevalence (for binary traits) between the score development population and the target population.*

Other minor comments:

1. On line 197, line 249 and line 532, a different style of citation seems to be used? (ref:[#] , instead of [#])

Thank you very much. We now have the correct citation style in the latest version of the manuscript.

2. For figure 4 top right, are the range inclusive or exclusive? E.g. for sample at 10 percentile, will they be grouped in [0-10%] or [10-20%]? Also, for multipaned plots, might be easier if the individual sub-plots are also labeled (e.g. 4a, 4b, 4c)

Thank you very much for pointing out the ambiguity in the notation. We used left-open intervals with the exception of the top 10%-tile bin. We have updated the figure. Thank you very much for your suggestion.

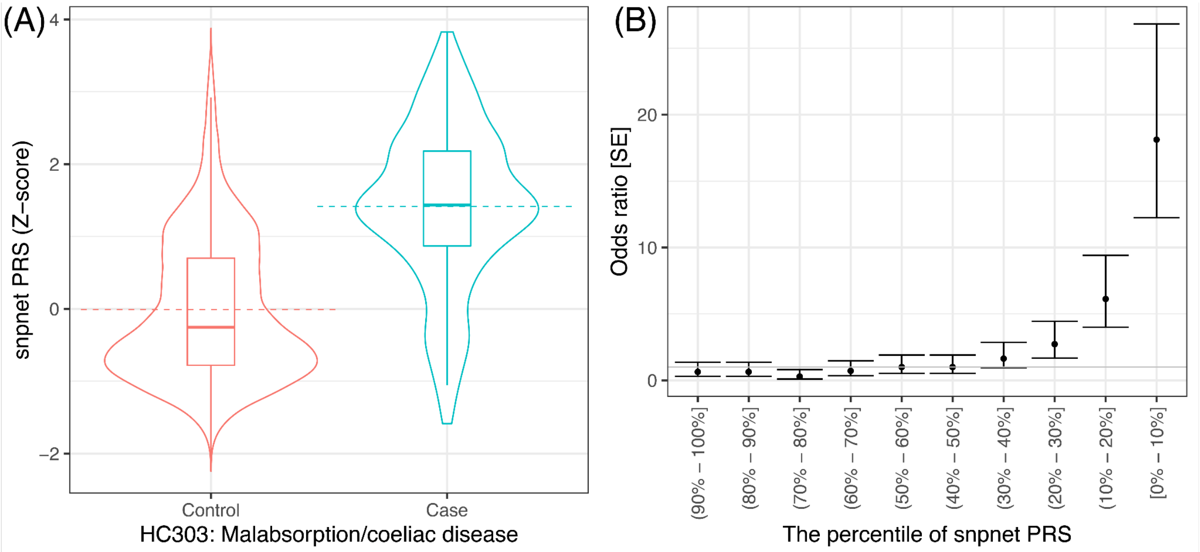

Figure 4. The sparse PRS model and their predictive performance for celiac disease. (A, B) the predictive performance of celiac disease PRS. (A) the celiac disease PRS distribution (y-axis) in a hold-out test set stratified by the disease case status (x-axis). The dashed lines represent the mean and the quantiles are shown as box plots. (B) The disease prevalence odds ratio compared to the individuals with middle (40-60th percentile) PRS score stratified by PRS percentile bins. The error bars represent standard error (SE).

Thank you very much for taking the time to review the manuscript and for providing detailed feedback.

Reviewer #2

The authors have addressed my comments, but the revision has introduced some strong statements in the discussion which I believe are scale and power dependent. Therefore, I have additional comments.

Thank you very much for taking the time to review the manuscript and for providing detailed feedback. We are confident that your comments have improved the clarity of the manuscript. Here are the responses to your suggestions.

New Figure 2A. For binary traits estimates of SNP-based heritability depends on proportion of GWAS discovery sample are cases, and Pseudo-R2 depend on the proportion of the target sample are cases. Although requiring a user-specified lifetime risk it would make more sense for these axes to be on the liability scale (even if lifetime risk used is the proportion of cases in the sample since all traits are in UKB) since then both axes are on the same scale and comparisons across traits are more valid.

Thank you very much for pointing out the fact that pseudo-R^2 depends on the case prevalence. While conversion of heritability and pseudo-R^2 to liability scale is an attractive suggestion, the population prevalence parameter is not available only for some of the traits and we are unaware of how to properly consider the potential ascertainment bias in the UK Biobank study.

In the previous version of the manuscript, we had Figure 2A comparing the estimated SNP-based heritability (in observed scale) against the predictive performance of the PRS models quantified by Tjur's pseudo-R^2 (in observed scale). Based on your feedback on the types of pseudo-R^2, we revised Figure 2A using Nagelkerke's pseudo-R^2.

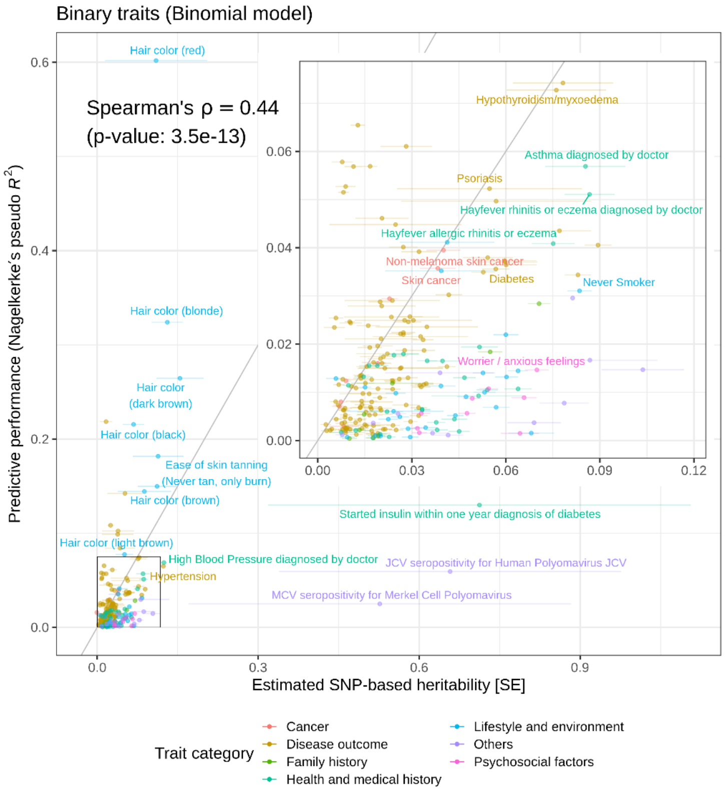

Figure 2A. Comparison of the estimated SNP-based heritability and predictive performance across the 813 traits with significant PRSs. The predictive performance (Nagelkerke's pseudo-R2 for 244 binary traits [left]) of the PRS models that only consider genetic variants are compared against the estimated SNP-based heritability. The solid gray lines represent y = x. We show the points on the bottom left corners in the inset plots. The error bars represent standard error.

When we take the case frequency in the UK Biobank as the estimate of the population case prevalence, we can convert the SNP-based heritability estimates and Nagelkerke’s pseudo-R^2 into liability scale. As you will see in the plot below, the liability-scale metrics, especially Nagelkerke's pseudo-R^2, for some traits (especially the ones with lower case counts in UK Biobank) showed very high value. For example, Iritis has a case count of 146 and 40 in the PRS model development set and hold-out test set, respectively.

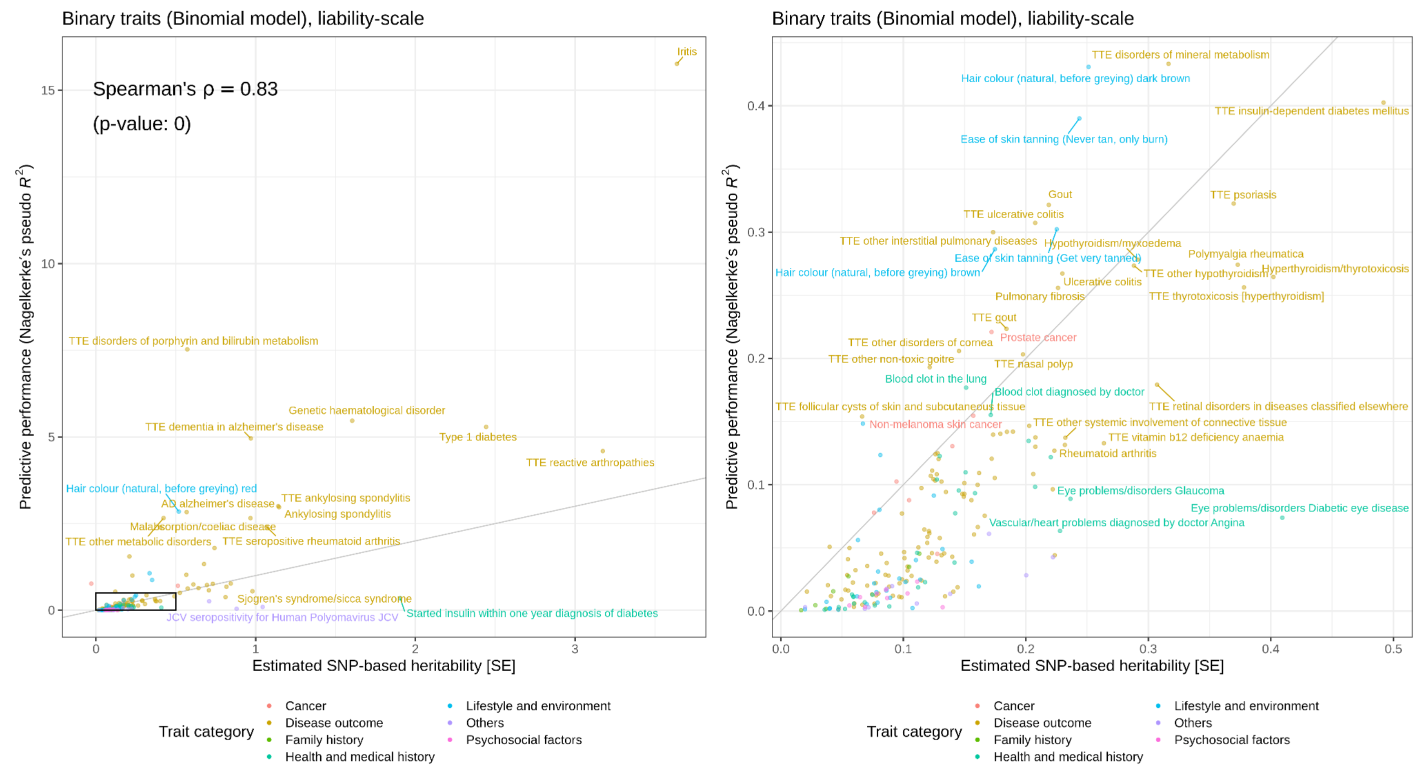

Figure. Comparison of the estimated SNP-based heritability (liability scale) and predictive performance (Nagelkerke’s pseudo-R^2 in liability scale) across the 244 binary traits with significant PRSs. The left panel shows all the 244 traits. A subset of traits marked with black box is shown in the right panel.

For the proper conversion to liability-scale metrics, we believe the reliable estimate of population prevalence of the binary traits would be helpful, but such parameters are not available for 244 traits (including both disease and non-disease traits) considered in the study. To avoid the potential confusion, we decided to show the results on the observed-scale. Related to this, reviewer #1 also asked if there are differences in the case frequency between the PRS score development set and PRS evaluation set. As you see in the plot below, we confirmed that there is no notable difference.

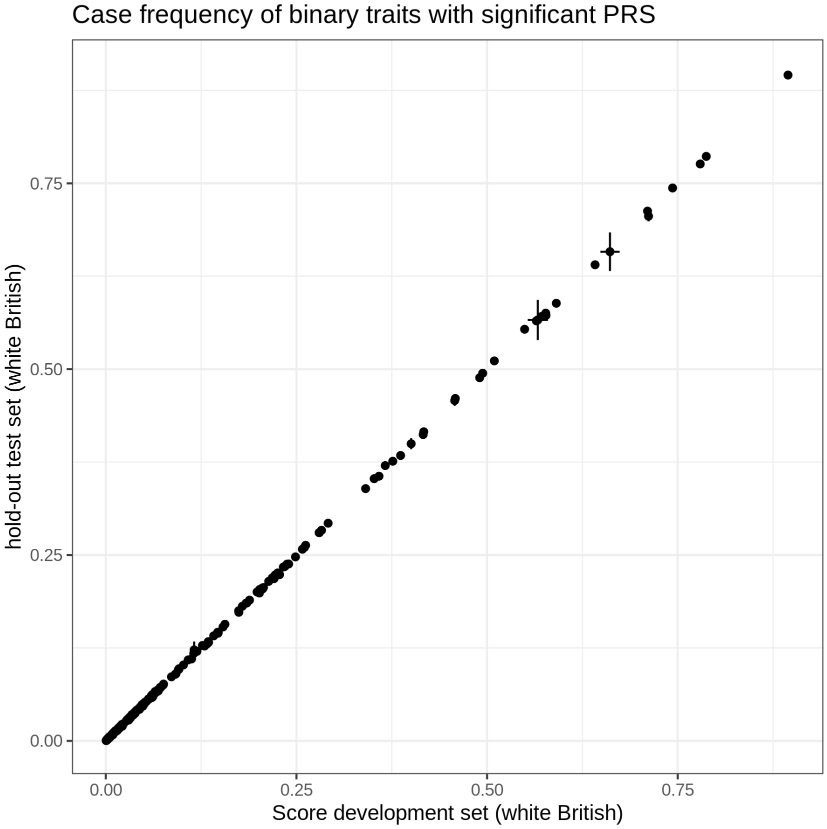

Figure. The case frequency difference between the score development set (training set and validation set, x-axis) and the hold-out test set (test set, y-axis). The error bars represent 95% confidence intervals.

With those observations, we now show the observed-scale heritability and observed-scale predictive performance (Nagelkerke's pseudo-R^2 and R^2 for binary and quantitative traits, respectively) in Figure 2. In the updated version of the manuscript, we clarified the presented SNP-based heritability estimates are the observed scale. We also acknowledged the advantage of the liability-scale heritability/pseudo-R^2 in the discussion.

Lines 195-202, Page 7, Results

> *We estimated the SNP-based heritability by applying linkage disequilibrium (LD) score regression (LDSC)[27] on genome-wide association study (GWAS) summary statistics. We compared it against the predictive performance (R^2 for quantitative traits and Nagelkerke's pseudo-R^2 for binary traits) of the significant PRS models (Fig 2). Across 244 binary traits and 569 quantitative traits with significant PRS models, we found higher estimated observed-scale heritability for quantitative traits. Overall, we found a significant correlation between the estimated SNP-based observed-scale heritability and*

predictive performance (Spearman's rank correlation coefficient ρ = 0.44, p-value = 3.5 x 10^{-13} for binary traits, ρ = 0.46, p-value = 1.4 x 10^{-31} for quantitative traits).

Lines 341-344, Page 13, Discussion

For binary traits, we used observed-scale pseudo-R^2 and observed-scale SNP-based heritability estimates, given that population prevalence is available for only a subset of binary traits considered in the present study. Conversion to liability-scale estimates will further enhance the validity of the comparison[35] and is of interest for future investigation.

References:

35. Lee SH, Goddard ME, Wray NR, Visscher PM. A better coefficient of determination for genetic profile analysis. Genet Epidemiol. 2012;36: 214–224. doi:10.1002/gepi.21614

Figure 5A and Figure 6 LHS use "incremental AUC". AUC has the nice property that it doesn't depend on the proportion of cases in the sample, both other than that it has very non-linear properties with respect to quantitative genetic metrics of polygenic traits such as heritability. For example, while a linear relationship might be expected in incremental R2 for quantitative traits (Figure 6 bottom left quadrant) I wouldn't expect a linear relationship in incremental AUC. This may impact the conclusion line 331 "we found a significant correlation across quantitative traits but not within binary traits" Suggest of these analyses R2 liability is used.

Thank you very much for raising this important point. In the revised manuscript, we now use Nagelkerke's pseudo-R^2 (observed-scale) as the primary metric to evaluate the predictive performance for binary traits. Indeed, when we use Nagelkerke's pseudo-R^2 (observed-scale), we observed the significant rank-based correlation between the incremental predictive performance and the number of active variables in PRS models for both binary and quantitative traits.

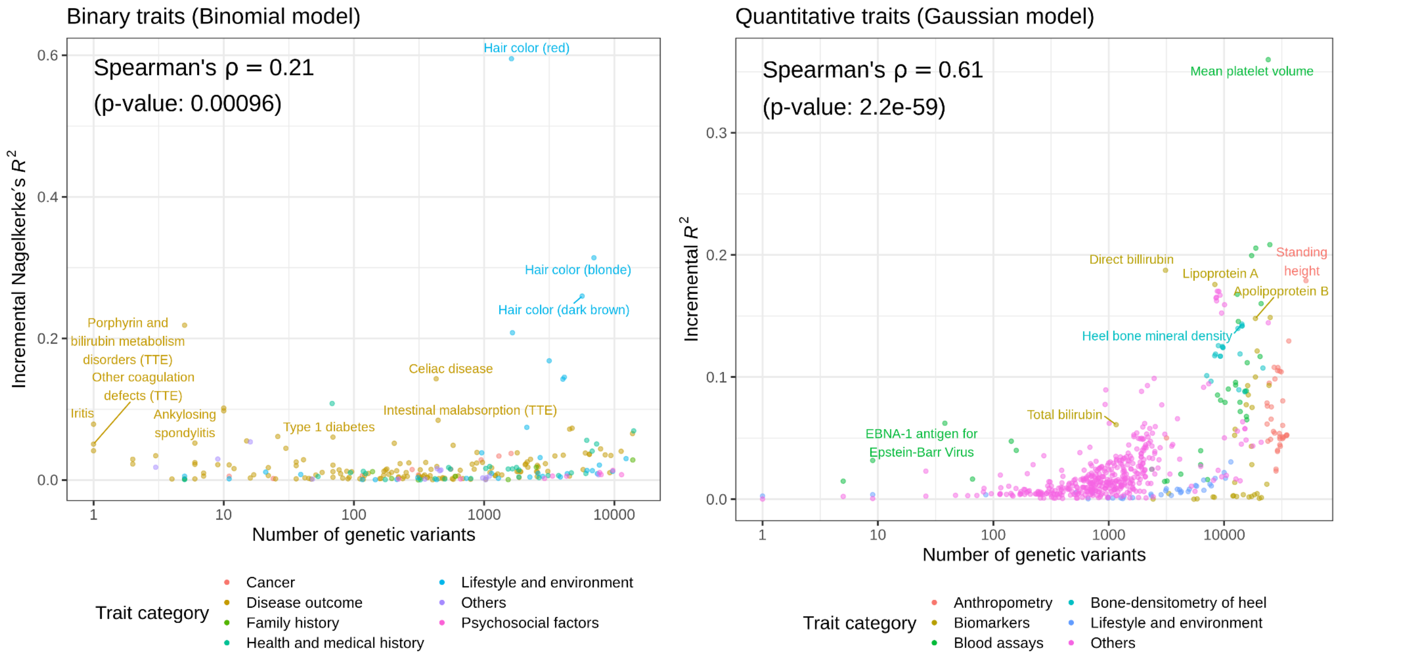

Figure 5. Comparison of the effect size and the model size of sparse PRS. The number of the genetic variants included in the model (size of the model, x-axis) and the incremental predictive performance (effect size of the model, y-axis) are shown for 244 binary traits (left) and 569 quantitative traits (right). TTE: time-to-event phenotype.

Following your suggestion, we now report the results of the transferability assessment using Nagelkerke's pseudo-R^2.

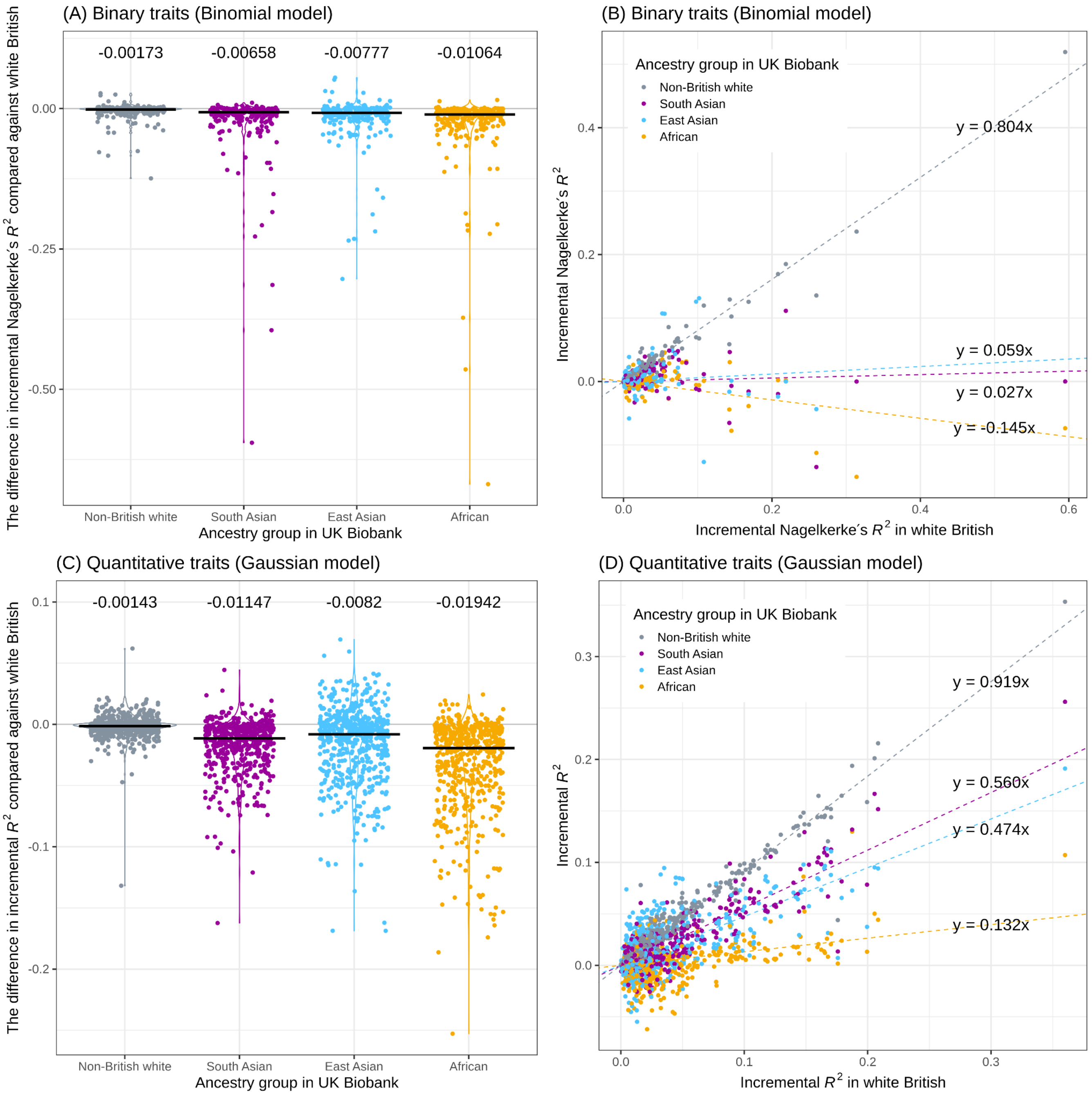

Figure 6. Transferability assessment of PRS models across ancestry groups in the UK Biobank. The incremental predictive performance (Nagelkerke's pseudo-R^2 for 244 binary traits (A, B) and incremental R^2 for 569 quantitative traits (C, D)) was quantified in individuals in different ancestry groups in the UK Biobank and was compared against that in the hold-out test set constructed from the individuals in white British ancestry group. (A, C) the difference in the incremental predictive performance between the target group (x-axis, double-coded with color) and the source white British cohort. The median values are shown as black horizontal bars and numbers. (B, D) comparison of the incremental predictive performance in the target group (color) and the test set. A simple linear regression fit was shown for each ancestry group with the dashed lines. The slopes of the regression lines were also shown.

The point being made here “While the underlying genetic architecture of binary traits may span the gamut of a wide variety of polygenicity, that of highly heritable quantitative traits may not be compatible with monogenic inheritance as illustrated in the wide adoption of Fisher’s infinitesimal model”. That is a very broad statement not really relevant to the study, suggest delete. Moreover, expressions of genes are quantitative traits that likely span the gamut of genetic architectures.

Thank you very much for pointing this out. We removed the sentence in the updated version of the manuscript.

I am concerned about the new conclusions that contrast binary traits with quantitative traits with only a nod to differences in power. It is intuitive that for the same N (ie UKB sample size) as the proportion of cases tends to zero the power of the sample for detection of association is reduced. I think Yang et al (2009) equation 3 could help quantify expectations doi:10.1002/gepi.20456

Thank you very much for raising important concerns and for pointing out the relevant literature, which is now cited as reference [31]. With Nagelkerke's pseudo-R^2, we now observe the significant correlation between the incremental predictive performance and the number of active variables in PRS models for both binary and quantitative traits. Nonetheless, we noted the power difference in binary traits and quantitative traits in the discussion.

Lines 269-274, Page 10, Results

> *We examined whether there is a relationship between the number of active variables in the significant PRS model and the incremental predictive performance. The significant correlation between the two quantities is stronger in quantitative (Spearman’s rank correlation coefficient ρ = 0.61, p = 2.2 x 10^{-59}) traits than in binary (ρ = 0.21, p = 9.6 x 10^{-4}), reflecting the difference in power between binary and quantitative traits[31].*

Lines 322-327, Page 13, Discussion

> *We assessed the effect size of the PRS model by quantifying the incremental predictive performance, which we define as the difference in the predictive performance between the covariate-only model and the full model consisting of both covariates and genetics. In both quantitative and binary traits, we find a significant correlation between the number of independent loci included in the model and their incremental predictive performance.*

Lines 350-356, Page 13, Discussion

> *Nonetheless, when we assess the incremental predictive performance across ancestry groups by comparing the full model consisting of the genetic data and basic covariates and the covariate-only model, we found the binary traits, including disease outcomes, have lower transferability compared to quantitative traits, including biomarkers, blood measurements, and anthropometric traits. The power difference between binary and quantitative traits[31], limitation in power for some traits, especially for*

> *the binary traits with limited case counts, and differences in heritability may be the contributing factors of the observed difference*

Supp Table 6 seems to have a column missing -across the labels in column A-D there are 3 sets of results. Model column? I have never seen TjurR2 presented before in this context. It is presented together with NagelkerkeR2. There is not justification as to why TjurR2 should be presented. Both I believe are dependent on the proportion of cases in the sample . Some of the AUC values seem implausibly high given the R2? Check?

Thank you very much for pointing this out. The model column was indeed missing in the previous version. We have now included the “model” column in the S6 table.

Tjur’s pseudo-R^2 is a recently proposed metric (Tjur T 2009) and is defined as the difference in the mean predicted probability between cases and controls and is closely related to the sum of squared residuals. While this has ease of interpretation, the metric is not based on the likelihood function and is not clear how one would convert it to a liability scale, given the population prevalence. Moreover, as you correctly pointed out, Tjur’s pseudo-R^2 is less consistent with ROC-AUC than Nagelkerke's pseudo-R^2.

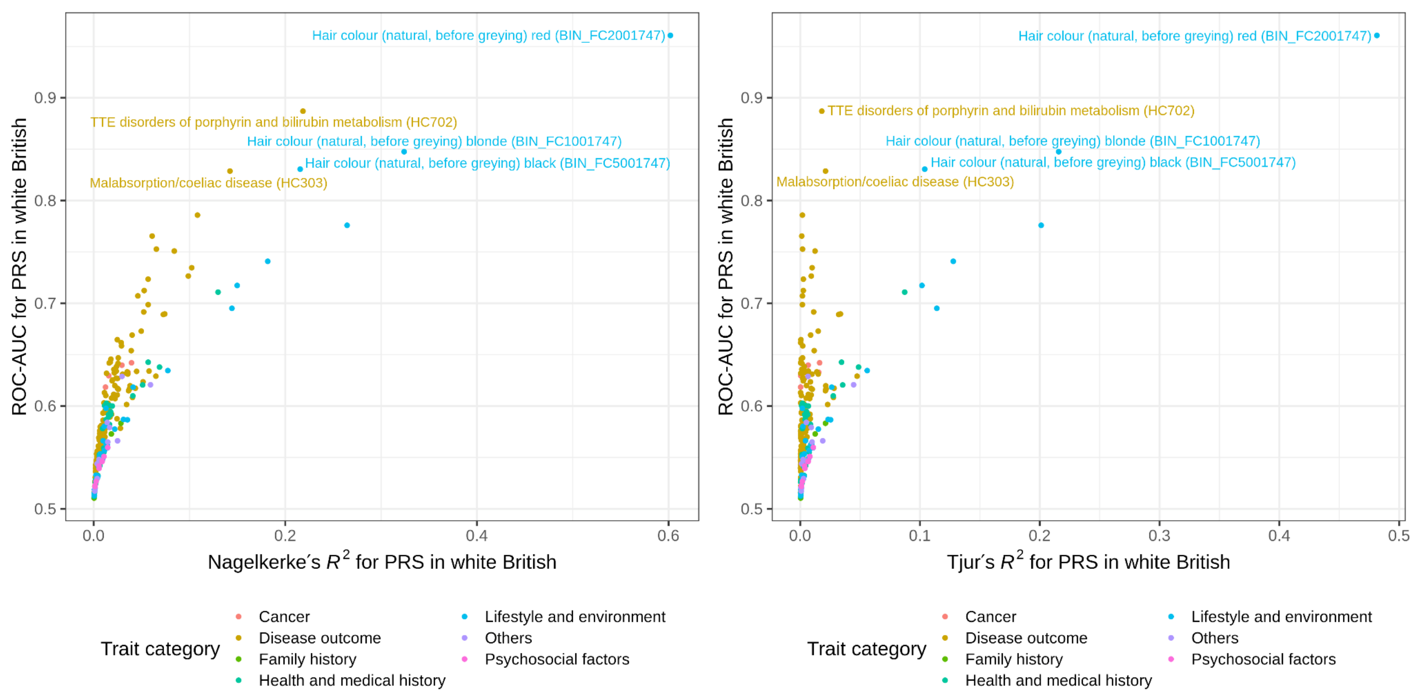

Figure. Comparison of Nagelkerke's pseudo-R2 vs ROC-AUC (left) and Tjur's pseudo-R2 vs ROC-AUC (right) for the predictive performance of PRS across 244 binary traits.

For those reasons, we now use Nagelkerke's pseudo-R^2 as the primary metric of evaluation. Tjur’s pseudo-R^2 is provided only in the supplementary table S6 (where we provide Nagelkerke's pseudo-R^2, Tjur’s pseudo-R^2, and ROC-AUC) as well as on Global Biobank Engine.

Thank you very much for taking the time to review the manuscript and for providing detailed feedback.

Reviewer #3

The additional analyses and explanations in this revision result in a much improved manuscript describing the phenome-wide application of BASIL to derive PGS in UKB. The authors have addressed all my concerns (especially with respect to the description of variant-penalties), the analyses are technically sound and well described.

Thank you very much for taking the time to review the manuscript and for providing detailed feedback.
